# Development and psychometric properties of the Clinical Anxiety Scale for People with Intellectual Disabilities (ClASP-ID)

**DOI:** 10.1186/s11689-024-09554-9

**Published:** 2024-07-27

**Authors:** Jessica Eliza Mingins, Joanne Tarver, Effie Pearson, Georgina Edwards, Megan Bird, Hayley Crawford, Chris Oliver, Lauren Shelley, Jane Waite

**Affiliations:** 1 School of Psychology, College of Health and Life Sciences, Aston University, Birmingham, B4 7 ET United Kingdom; 2 School of Health and Care, Coventry University, Priory St, Coventry, CV1 5FB England; 3 Mental Health and Wellbeing Unit, Warwick Medical School, University of Warwick, Coventry, CV4 7AL England; 4 School of Psychology, University of Birmingham, Edgbaston, B15 2TT England

**Keywords:** Anxiety, Intellectual disability, Autism, Mental Health, Measure development, Questionnaire, Assessment, Pain

## Abstract

**Background:**

There is a critical need for the development of dependable and valid anxiety assessment tools suitable for people with moderate to severe intellectual disabilities, particularly those who speak few or no words. Distinguishing anxiety from distress caused by physical discomfort (pain) or characteristics associated with autism, prevalent in this population, necessitates specialised assessment tools. This study (a) developed a parent-report anxiety questionnaire tailored for individuals with severe to moderate intellectual disabilities, potentially with a co-diagnosis of autism, and (b) evaluated the psychometric attributes of this novel measure.

**Methods:**

A comprehensive approach involving literature reviews, inspection of existing tools, and interviews with clinicians and parents guided the creation of the Clinical Anxiety Scale for People with Intellectual Disabilities. The tool was completed by parents or caregivers (*N* = 311) reporting on individuals aged 4 or older with intellectual disabilities.

**Results:**

Exploratory factor analysis indicated a four-factor structure encompassing anxiety, pain, low energy/withdrawal, and consolability. The anxiety factor explained the most variance in scores (26.3%). The anxiety, pain, low energy/withdrawal subscales demonstrated robust internal consistency (α = 0.81-0.92), and convergent, divergent, and discriminant validity. Robustness of these subscales was further evidenced by test-retest reliability (ICC = 0.79-0.88) and inter-rater reliability (ICC = 0.64-0.71). Subgroup analyses consistently demonstrated strong psychometric properties among individuals diagnosed with non-syndromic autism (*N* = 98), children (*N* = 135), adults (*N* = 175), and across diverse communication abilities within the sample. Moreover, individuals diagnosed with both autism and anxiety exhibited significantly higher scores on the anxiety subscale compared to those without an anxiety diagnosis, while showing no difference in autism characteristic scores.

**Conclusions:**

The findings indicate that the Clinical Anxiety Scale for People with Intellectual Disabilities is a promising measure for use across diverse diagnostic groups, varying communication abilities, and with people with moderate to severe intellectual disabilities.

**Supplementary Information:**

The online version contains supplementary material available at 10.1186/s11689-024-09554-9.

## Background

### Anxiety in intellectual disabilities

Anxiety is more prevalent among individuals with intellectual disabilities, ranging from 14 to 42%, compared to the 2–24% rate in the general population [1–4]. This elevated anxiety occurrence is influenced by the aetiology of intellectual disabilities, with certain rare genetic syndromes demonstrating higher anxiety rates than mixed aetiology groups [5–7]. Despite being individually rare, these genetic syndromes collectively represent a significant proportion of people with severe intellectual disabilities, which may heighten anxiety risk within this population [8–10]. Moreover, many individuals with severe intellectual disabilities have co-occurring autism^1^ (lifetime prevalence: 42%), which is a further risk factor for anxiety [13–15].

Anxiety significantly impacts the well-being of individuals with intellectual disabilities, as well as the general population. It leads to adverse outcomes such as depression, disrupted sleep patterns, compromised social functioning, and diminished quality of life [16–19]. However, despite concerns regarding anxiety risk within people with moderate to severe intellectual disabilities, and the consequential effects of anxiety, there remains a significant scarcity of assessment measures tailored for individuals in this population [20]. This dearth of measures severely limits research aimed at quantifying, understanding, and addressing anxiety in this population.

### Challenges with anxiety measurement in intellectual disabilities

Measuring anxiety in individuals with moderate to severe intellectual disabilities, especially those who speak few or no words, presents several challenges. First, anxiety assessment is complicated by the overlap between behavioural indicators of anxiety, distress due to physical health issues (i.e., pain), or the presence of alternative diagnoses (e.g., autism or depression). For instance, negative vocalisations like screaming and crying, sleep disturbances, and behaviours that challenge are commonly associated with pain, anxiety, and low mood [21–25]. Furthermore, psychomotor agitation, such as restlessness and irritability have been associated with both anxiety and low mood in people with intellectual disabilities [22, 26]. Differentiating anxiety from other possible causes of presenting behaviour is crucial, as mental health difficulties require a different approach to intervention compared to distress arising from physical health difficulties. Alternatively, a multi-modal intervention may be required where there is an interaction between factors leading to distress. The importance of disentangling anxiety from indicators of pain, and raising awareness among clinicians and researchers of the need to consider differential diagnosis, is paramount given the high rates of painful physical health conditions experienced by people with moderate to severe intellectual disabilities [27–29].

A further challenge is that qualitative differences in anxiety presentation in people with intellectual disabilities have been reported compared to the general population. For example, repetitive behaviours have been associated with anxiety in people with intellectual disabilities and autistic people [30–32]. Including the full range of potential behavioural markers of anxiety during the development of measures may be crucial for detecting anxiety in people who speak few or no words who cannot communicate their anxiety verbally. Despite this, atypical behavioural indicators of anxiety can be overlooked or misattributed to intellectual disabilities, a person’s genetic syndrome, autism, or other co-occurring conditions, a phenomenon known as diagnostic overshadowing [21].

There are no anxiety measurement tools that were developed with the aim of thoroughly addressing these challenges. In the absence of available measures, researchers and clinicians often rely on anxiety measures developed for the general population. However, many of these measures depend on a person being verbal and able to describe worries or label their internal states. Parents and caregivers reporting on anxiety in their children with intellectual disabilities may be less likely to endorse items regarding their child’s internal thoughts and feelings, leading to the under-reporting of anxiety symptomatology [33]. Hence, these measures are not appropriate for people with severe intellectual disabilities.

Some questionnaire measures designed to assess anxiety in people with intellectual disabilities do exist, such as the Anxiety Depression and Mood Scale (ADAMS; 34) and the Diagnostic Assessment for the Severely Handicapped (DASH-II; 67). The ADAMS has robust psychometric properties, however, its suitability for people with severe intellectual disabilities is uncertain as it was developed for those with mild to moderate intellectual disabilities [20, 34]. The DASH-II, while a reliable and valid measure developed for people with severe intellectual disabilities, was partly informed by the diagnostic criteria from the Diagnostic and Statistical Manual of Mental Disorders (3rd ed., rev., DSM-III-R) [35], which may not be appropriate when applied to people with intellectual disabilities [22, 36]. Furthermore, while the DASH-II was a pioneering measure developed in the 1990s, the recent developments in the understanding of the anxiety profile observed in autistic people with intellectual disabilities necessitates further measure development [37]. Finally, there is a need for a measure that uses contemporary terminology and that prompts clinicians and researchers to consider differential diagnosis (e.g., pain) when behavioural indicators of distress are observed [38].

Some promising new measures have been developed to examine anxiety experienced by autistic people, such as the Anxiety Scale for Children with Autism Spectrum Disorder (ASC-ASD) [39] and the Anxiety Disorders Interview Schedule-Autism Addendum (ADIS/ASA) [40]. Both measures perform well but were developed specifically for people without intellectual disabilities or with a mild intellectual disability. Therefore, there remains a dearth of measures designed to assess anxiety that are appropriate for people with moderate to severe intellectual disabilities, particularly those who speak few or no words and those with co-occurring autism [20].

Flynn et al. [20] advocate for the integration of bottom-up approaches in the development of measures tailored for individuals with severe intellectual disabilities. They suggest reducing reliance on conventional diagnostic criteria and existing measures developed for people without intellectual disabilities. Such an approach is justified given the anxiety-related behaviours shown by people with severe intellectual disabilities may elude capture by current assessment tools [6]. Thus, adopting a bottom-up approach, which draws insights from detailed behavioural descriptions of anxiety, is imperative for the development of new measures specifically designed for individuals with moderate to severe intellectual disabilities.

### Aims

The present study had three aims:

1. To draw on a range of approaches, including bottom-up methodology, to develop an informant-report anxiety screening measure that is suitable for people with moderate to severe intellectual disabilities, including those who speak few or no words and individuals with a diagnosis of autism.
2. To develop a measure to overcome limitations of previous measures by having the potential to inform differential diagnosis between anxiety and other forms of internal distress, namely pain and low mood.
3. To investigate the psychometric properties of the newly developed measure, including the factor structure, internal validity, test-retest reliability, inter-rater reliability, convergent and divergent validity.

## Method

The study has received a favourable ethical decision from the NHS Research Ethics Committee Wales REC 3 (ref: 18/WA/0139); Research Registry (UIN: researchregistry5086).

### Development of items for the clinical anxiety scale for people with intellectual disabilities (ClASP-ID)

#### Literature review

Due to the complexity of differentiating anxiety in people with intellectual disabilities from other forms of internal distress (pain and low mood) a broad and inclusive approach was taken to item generation. Systematic literature reviews on the behavioural indicators of anxiety and low mood in intellectual disabilities were conducted [22, 41]. Behaviours were extracted from these reviews and entered onto a database for the purpose of item development. To generate additional behavioural items to those identified by the systematic reviews, thirty-six existing measures pertaining to measure anxiety, low mood and/or pain within typically developing children (e.g., Spence Children’s Anxiety Scale, Face, Legs, Activity, Cry, Consolability scale), autistic individuals (e.g. Autism Spectrum Disorder -Comorbidity for Adults) and individuals with mild to moderate intellectual disabilities (e.g. ADAMS, Glasgow Anxiety Scale), were examined, along with existing measures of mental health and pain for individuals with severe to profound intellectual disabilities and individuals who speak few or no words (e.g. Mood Interest and Pleasure Questionnaire; see additional file 1 for full list). These 36 measures were identified from the systematic reviews of behavioural indicators of anxiety and low mood [22, 41–43] and existing reviews of mental health tools and pain indicators in people with intellectual disabilities and autism [20, 44].

Across these measures 1028 items were reviewed. To aid item generation and reduction, these items were grouped into categories based on topographical similarity. All items and item categories were reviewed by three members of the research team (JT, GE and JW).

#### Interviews

To further supplement the item database, and due to the complexity of identifying anxiety in the intellectual disability population, parent interviews were conducted to gather information on individual anxiety signatures displayed by children and adults with intellectual disabilities; published in [38]. Parent strategies to manage anxiety were discussed as part of these interviews to provide insight into mutually reinforcing behaviour that may indicate anxiety (e.g., avoidance of feared situations; published in [25]. The interview schedule was previously developed as part of a research project aiming to explore anxiety in intellectual disabilities utilising clinical formulation frameworks [45]. In total, 30 parent/carer interviews were conducted with parents of people who speak few or no words. This included 21 autistic children and adults with no known genetic condition, in addition to 9 people with intellectual disabilities of mixed aetiology (e.g. fragile X syndrome, Cornelia de Lange syndrome) to pick up wider anxiety phenomenology (3/9 also had a diagnosis of autism). For the purposes of tool development and item generation, the interviews were coded using an existing coding proforma to identify behaviours and triggers for anxiety [45]. There were few differences between reported anxiety signatures and triggers between children and adults [38]. See additional file 2 for a summary of coded interview data.

Nine healthcare workers from NHS learning disability clinics (clinical psychologist *n* = 2; community learning disability nurse *n* = 2; clinical nurse specialist *n* = 2; paediatric neuropsychiatrist *n* = 1; psychiatrist *n* = 1; paediatrician *n* = 1) were also interviewed about the presentation of anxiety in people with intellectual disabilities, differential diagnosis (e.g., pain, low mood, autistic characteristics) and the implications for developing assessment measures (for full details see 38).

#### Beta version of the measure

The information generated from these steps was synthesised, and items were drafted to represent the identified behavioural codes. This resulted in a 69-item beta version of the scale that captured the breadth of behavioural expression identified in the interviews and literature reviews (see additional file 3). The beta version also included one additional open-ended question to ask parents about other changes in mood or behaviour not captured in the questionnaire. Due to the processes followed above, it was anticipated that the 69-items would assess behaviours associated with anxiety, low mood, and pain in people with intellectual disabilities. Due to previous studies indicating overlapping behavioural indicators across anxiety, low mood and pain, no a-priori assumptions were made about which behaviour items would load onto which specific scales, as this was to be examined via the exploratory factor analysis.

The 69 items of the ClASP-ID items were measured on a 7-point Likert scale from ‘almost never’ to ‘all the time’ or ‘almost never’ to ‘more than once a day’. Research has demonstrated several benefits of the 7-point scale including it providing sufficient granularity, leading to more nuanced and accurate data, reducing midpoint bias, and providing balanced response options [46–48]. In line with methodological guidance, each point on the scale was operationalised, to reduce need for individual interpretation of the response options [47, 49]. Respondents are asked to report on the frequency of behaviours over the last one month.

Following clinician feedback of the importance of being able to assess a person’s change from baseline, particularly on items which overlapped with autistic characteristics (e.g., repetitive behaviour; [38], 14 items included follow-up questions which asked whether behaviour is currently occurring more, at the same rate, or less than is typical of the person with intellectual disability. These questions were designed to provide clinical context and so were not included in the scoring of the questionnaire and therefore the psychometric validation.

#### Parent and clinician review of the 70-item scale

As a final step, the beta version of the measure was circulated to four parents of children with intellectual disabilities. All parents were part of the Cerebra Centre advisory panel. Due to the length of the draft measure and to reduce participant burden, the feedback process was conducted using a questionnaire feedback proforma (see additional file 4). Parents were asked to read the questionnaire instructions and provide feedback on elements of the instructions that were unclear. Parents then read each item of the questionnaire and were asked to note next to the item if it was confusing or unclear and suggest any alternatives for how a question could be phrased. Following feedback from the families, a panel of researchers (JW, JT & GE) discussed the comments, and final amendments were made to the questionnaire.

### Psychometric properties of the ClASP-ID

#### Recruitment

In total, 314 parents or caregivers of people with intellectual disabilities were recruited for the refinement and psychometric evaluation of the measure. When asked how they heard about the study, parents reported it was via invite from participating NHS trusts (*n* = 32), mailing lists of people who had previously taken part in research (*n* = 203), snowball sampling (*n* = 6) or social media (*n* = 18); some parents reported multiple recruitment routes and some declined to answer this question. Parents or caregivers were asked to complete study questionnaires about a person aged 4 or older with a diagnosis of an intellectual disability. There was no upper age limit, and no exclusion criteria based on diagnosis of co-occurring conditions such as rare genetic syndromes, neurodevelopmental conditions, physical or mental health difficulties. The minimal exclusion criteria facilitated a representative sample, given the high prevalence of co-occurring conditions in people with intellectual disabilities.

#### Participants

Of the 314 parents and caregivers who were recruited, three were excluded from the present analysis. This was because inclusion criteria necessitated a diagnosis of an intellectual disability, either by parent report or by scores on the Wessex Questionnaire [50]. The Wessex Questionnaire acts as a proxy measure for degree of disability (see measures section for more information). These three participants were excluded as they did not report that the person they care for had an intellectual disability *and* the scores provided on the Wessex Questionnaire were not consistent with the presence of an intellectual disability diagnosis. A further ten parents and caregivers did not report that the person they care for had a diagnosis of an intellectual disability, but scores on the Wessex questionnaire indicated that the person had communication or self-help needs consistent with the presence of intellectual disabilities. These ten participants were included in the analysis.

In total, 311 parents and caregivers were included in the analysis. They reported on a group of people with intellectual disabilities who ranged in age from 4 to 83 (M = 21.6, SD = 12.0) and were 61.4% male. Owing to the broad inclusion criteria, a range of rare genetic syndromes associated with intellectual disabilities were reported including Angelman syndrome (*n* = 45, 14.5%), fragile X syndrome (*n* = 26, 8.4%), and Cornelia de Lange syndrome (*n* = 22, 7.1%). Neurodevelopmental conditions were also commonly reported, including autism (*n* = 156, 50.2%) and ADHD (*n* = 44, 14.1%). Parents and caregivers reported a diagnosis of an anxiety disorder in approximately one fifth of the people with intellectual disabilities (*n* = 68, 21.9%). Parents and caregivers most frequently reported that the person they care for spoke odd words only (*n* = 127, 40.8%), was partly able in terms of self-help ability (*n* = 119, 38.3%) and fully mobile (*n* = 187, 60.1%). For full demographic information, see Table 1.

**Table 1:** Demographic characteristics of the sample

	N (%)
Person with ID gender	
Male	191 (61.4)
Female	120 (38.6)
Age of person with ID	
Under 18	135 (43.4)
18–24	75 (24.1)
25–34	49 (15.8)
35–44	38 (12.2)
45–54	11 (3.5)
55+	3 (1.0)
Intellectual Disability Diagnosis	
Yes	301 (96.8)
No	10 (3.2)
Syndrome Diagnosis	
Angelman Syndrome	45 (14.5)
Cornelia de Lange Syndrome	22 (7.1)
Fragile X Syndrome	26 (8.4)
Prader-Willi Syndrome	16 (5.2)
Down Syndrome	15 (4.8)
Cri du Chat Syndrome	11 (3.5)
Potocki-Lupski Syndrome	11 (3.5)
Tuberous Sclerosis	10 (3.2)
9q34 Deletion	8 (2.6)
Phelan McDermid Syndrome	6 (1.9)
Pitt Hopkins Syndrome	5 (1.6)
Other	44 (14.2)
Psychiatric and/or Neurodevelopmental Diagnosis	
Autism	156 (50.2)
Anxiety	68 (21.9)
ADHD	44 (14.1)
Obsessive-Compulsive Disorders	27 (8.7)
Depression	20 (6.4)
Verbal Ability^a^	
Non-verbal	81 (26.0)
Odd words only	127 (40.8)
Fully verbal	89 (28.6)
Can talk but doesn’t	14 (4.5)
Self-Help^a^	
Not able	112 (36.0)
Partly able	119 (38.3)
Able	80 (25.7)
Mobility^a^	
Non-ambulant	41 (13.2)
Partly mobile	82 (26.4)
Fully mobile	187 (60.1)

^a^ Information obtained using the Wessex Questionnaire [50]

#### Procedure

Information about the study was sent to participants via post, email or telephone to invite them to take part. The information sheets and study consent forms were available to participants on the survey engine Qualtrics. Once consent forms were completed, participants could proceed to complete study questionnaires. Parents and caregivers could also request paper copies of the information sheets, consent forms and study questionnaires if they preferred. Parents and caregivers completed an online battery of questionnaires on anxiety, pain, health difficulties, mood, autistic characteristics, intolerance of uncertainty, restricted and repetitive behaviours, behaviours that challenge and sensory processing differences. The battery of questionnaires was conducted as part of a wider project whereby the validation of the ClASP-ID was one aim of this project.

At the end of the questionnaire battery, parents and caregivers were asked if they were happy to be contacted in 2–4 weeks to complete portions of the battery for a second time to conduct an analysis of test-retest reliability. This data was available for 78 participants (see additional file 5 for demographics) and there were no significant differences in age, gender, ability level, and proportion of participants with an autism, anxiety or depression diagnosis between the test-retest reliability sample and the total sample. Participants were also asked if there was anyone else who knew the person they care for well who would also be willing to complete a small portion of the questionnaires to obtain a measure of inter-rater reliability. A second rater was available for 20 completions of the ClASP-ID (see additional file 5 for demographics). There were no significant differences in age, gender, ability level, and proportion of participants with an autism, anxiety or depression diagnosis between the inter-rater reliability sample (*n* = 20, 6% of sample) and the total sample.

Participants who did not complete the full questionnaire battery or had missing data were contacted once by the research team to complete their dataset. Where missing data remained, available-case analysis was used to preserve as much data as possible.

#### Measures

In the interest of brevity, only measures included in the present analysis, for the purpose of refinement and evaluation of the psychometric properties of the ClASP-ID, are described below.

##### Measure of interest- clinical anxiety scale for people with intellectual disabilities (ClASP-ID)

Parent and caregivers were asked to complete the beta version of the questionnaire (described above) which asks about behavioural indicators pertaining to anxiety, low mood and pain in people with intellectual disabilities. Items are scored on a seven-point scale based on frequency of behaviours.

##### Wessex questionnaire [50]

This is a 16-item informant report questionnaire designed to assess ability level including communication, self-help skills and mobility, used in this study as a proxy measure of intellectual disability. Presence of difficulties with communication, self-help skills such as washing and feeding, and mobility are consistent with diagnosis of intellectual disability [51]. Items are scored on a three-point scale, except for an item on speech which includes a fourth option to encompass those who can speak but do not. Higher scores on items indicate higher ability levels. Inter-rater reliability of the speech, self-help and mobility subscales are good at 82%, 78% and 92% respectively. This measure has been used extensively in large-scale questionnaire studies as a proxy measure of ability level, where in-depth assessments are not feasible [52–54].

##### Anxiety, depression and mood scale (ADAMS; 34)

The ADAMS is a 28-item informant report screening questionnaire for psychiatric disorders in intellectual disabilities. Items are scored on a four-point scale based on interference with daily life. Items are summed to create a total score and five subscales including general anxiety, social avoidance, depressed mood, manic/hyperactive, and obsessive/compulsive. The ADAMS has good internal validity (0.75–0.83), excellent test-retest reliability (0.81) and fair inter-rater reliability (0.48) [34]. The general anxiety subscale and the depressed mood subscales from the ADAMS were used in the ClASP-ID validation study given they capture the constructs of interest (anxiety and low mood), whereas the ClASP-ID was not developed to measure manic/hyperactive and obsessive/compulsive behaviours.

##### Health questionnaire- current [55]

The health questionnaire (current) is a 16-item informant questionnaire used to assess the presence and severity of health difficulties in the past month. Items are scored on a four-point scale and are summed to create an overall indicator of physical health in the past month. Inter-rater reliability of the measure is good (ICC = 0.71). The health questionnaire was used as a proxy measure of physical discomfort due to concerns that existing pain measures included items that could also be measuring anxiety, autism, or low mood.

##### Social communication questionnaire- current (SCQ; 68)

The SCQ (current) is a 40-item informant report questionnaire designed to assess behaviours associated with autism, including reciprocal social interaction, communication, and restricted and repetitive behaviours. Items are scored yes or no, and higher scores indicate higher autism characteristics. The SCQ is a sensitive screener for autism and has good internal consistency [56, 57].

#### Analytical approach

All the primary 69 items on the ClASP-ID were included in the analysis.

To refine the measure and assess the factor structure, an exploratory factor analysis (EFA) was conducted using principal axis factoring, with a promax rotation applied to account for the anticipated correlations between items and emerging factors. The sample size of 311 was above the minimum of 100 which is often cited as a requirement to conduct an EFA, however, it is slightly below the recommended five times the number of items recommended to conduct an EFA [58–61]. Overall, an EFA was deemed appropriate as statistical tests indicated the sample was appropriate; Bartlett’s test of sphericity was significant, and sampling adequacy was above the recommended 0.5 (KMO = 0.91; [61]). The EFA was repeated several times to obtain a simple structure, whereby every item loaded significantly to only one factor [62].

Internal consistency of the measure was established using Cronbach’s alpha, and test-retest and inter-rater reliability were established using intraclass correlations at scale level. Convergent and divergent validity was established by conducting Spearman’s rho correlations between the ClASP-ID subscales and the ADAMS and health questionnaire. Known groups validity was established by conducting t-tests to establish if those with a reported anxiety disorder diagnosis would score significantly higher on the ClASP-ID anxiety scale than those without an anxiety disorder diagnosis. This analysis was repeated in an autistic group only, to ensure the measure performed well in autistic populations. All data was analysed using SPSS 28.0.

## Results

### Exploratory factor analysis

An exploratory factor analysis (EFA, *N* = 304) was conducted using principal axis factoring, and a promax rotation was applied to account for the anticipated correlations between items and emerging factors [61]. All items were included in the EFA, regardless of their psychometric properties with the view to refine by factor loadings and psychometric properties later. This approach is common in the literature, for example in the development of the Anxiety Scale for Children with Autism Spectrum Disorder (ASC-ASD; 40).

The initial EFA extracted 17 factors. The first factor explained 26.3% of the variance, whilst the second, third and fourth factors explained a further 4.7%, 3.5% and 2.9% respectively. The remaining 13 factors explained fewer than 2% of the variance each and had few items loading to them. A four-factor solution was retained based on:

1. The percentage of variance explained by each factor, as described above.
2. Visual examination of the scree plot.
3. Clinical judgement of how items loaded to each factor, which was reviewed by a clinical psychologist and senior researcher with experience of mental health assessment.

The EFA was run for a second time, this time forcing a four-factor solution. At this stage, the ClASP-ID was refined, and several items were removed. This included: items which did not load to the final four factors (23 items), items with dual loadings above 0.4 (1 item), items with poor test-retest reliability of below 0.4 by Spearman’s correlation (1 item), items where the Cronbach’s alpha of the scale improved when removed (1 item), and items which could not distinguish between a those with and without a diagnosis of an anxiety disorder, a high and low pain group, and those with and without a diagnosis of depression by t-test (8 items). For a list of items which were removed from the ClASP-ID, see additional file 3.

The EFA was run for a third and final time with only the 35 remaining items, again forcing a four-factor solution. Two items no longer loaded to any of the four factors and were subsequently removed. Therefore, the final scale contained 33 items across four factors. Please see additional file 3 for a full list of items removed from the ClASP-ID. With the input of two clinical psychologists, the first, and largest, factor which contained 14 items was labelled anxiety, the second factor contained 9 items and was labelled pain, the third factor contained 6 items and was labelled low energy/withdrawal (a potential proxy measure of depression/low mood), and the final factor contained four items and was labelled consolability. Please see Table 2 for the final factor structure of the ClASP-ID. 

**Table 2:** Factor structure of the ClASP-ID

Item	Factor 1 Anxiety	Factor 2 Pain	Factor 3 Low Energy/ Withdrawal	Factor 4 Consolability
68. We are unable to do ‘typical’ day to day activities because of the emotional distress that would cause him/her (e.g. holidays, visiting friends, going for meals, general days out)	0.779			
23. Does he/she ever look very worried or anxious?	0.775			
69. We are unable to do activities we used to do with the person I care for because of the emotional distress he/she would experience	0.768			
7. Does he/she appear on edge OR on the look out for danger?	0.752			
32. Over the past month, have you noticed his/her face look tense?	0.687			
25. Does he/she have an angry look on his/her face?	0.680			
12. Does he/she appear restless or agitated?	0.671			
13. Does he/she ever run away or hide from certain objects or situations?	0.631			
20. Does he/she avoid (or try to avoid) certain objects or places?	0.628			
6. Does he/she ever make negative or frustrated vocalisations? (e.g. whining, grumbling, growling, shouting, screaming)	0.590			
14. Does he/she ever cover him/herself with a blanket or try to place a barrier between him/herself and others or a situation?	0.550			
11. Does he/she pace around the room?	0.523			
26. Does he/she startle easily, or easily alarmed?	0.520			
17. Does he/she ever freeze suddenly (stick to the spot) in response to specific situations?	0.457			
35. Over the past month, has his/her movements ever become jerky?		0.686		
28. Over the past month, have you noticed increased or different leg movements? (e.g. restlessness, tense, tremors, kicking, drawing legs up, jerking)		0.647		
10. Does he/she ever seem protective of a particular part of his/her body? (e.g. holding it, guarding it, flinching? )		0.566		
21. Does he/she ever take sharp intakes of breath or gasp?		0.543		
36. Over the past month, has his/her lips ever become tight, pout or quiver?		0.537		
16. Does he/she ever have watery eyes that is different from crying?		0.494		
19. Does he/she ever grind his/her teeth?		0.476		
31. Over the past month, have you noticed that he/she shakes or trembles?		0.424		
34. Over the past month, has he/she been hitting, holding or touching a part of their body?		0.410		
52. Does he/she lack energy?			0.795	
53. Does he/she get tired for no apparent reason?			0.737	
4. Has he/she seemed withdrawn with ‘vacant’?			0.618	
60. Is he/she spending more time asleep than usual? (e.g. not waking in the morning, sleeping during the day)			0.566	
45.Has he/she lost interest in activities that he/she used to enjoy?			0.434	
61. Is he/she quiet and spending time alone?			0.430	
66. When the person I care for is distressed, I am able to calm or comfort him/her				0.668
67. When in certain preferred environments (e.g. home, their bedroom) the person I care for generally appears calm and relaxed				0.627
65. Removing the person I care for from a situation, or removing an item/object generally calms them down				0.476
64. Preparing him/her before things happen helps to reduce his/her distress				0.464

### Reliability

#### Internal consistency

Internal consistency (*N* = 309) of the anxiety subscale was excellent (α = 0.92) and the pain and low energy/withdrawal subscales were good (α = 0.81, and α = 0.81). Cronbach’s alpha for the consolability subscale was lower (α = 0.63). Cronbach’s alpha was also calculated for several sub-groups to ensure performance was maintained across the sample. This included: children only (*n* = 135), adults only (*n* = 175), autistic groups (*n* = 156 all autism, *n* = 98 non-syndromic autism), and those with the highest level of support needs (*n* = 211 minimally verbal, n = 81 non-verbal, *n* = 123 non- or partially ambulant, *n* = 105 minimally verbal *and* not able in terms of self-help skills). For all these groups, internal consistency of the anxiety, pain and low energy/withdrawal subscales remained excellent or good. The Cronbach’s alpha of the consolability subscales remained consistent in most groups but improved in others (α = 0.58-0.64). Please see additional file 6 for a full breakdown of internal consistency in these sub-groups.

#### Test-retest reliability

Test-retest reliability at 2–4 weeks (*N* = 78) was good for the anxiety, pain and low energy/withdrawal subscales (ICC = 0.88, 0.80 and 0.79 respectively), and moderate for the consolability subscale (ICC = 0.67). Where sample sizes allowed, test-retest reliability was calculated for the same sub-groups in the sample as defined above. Test-retest reliability of the anxiety, pain and low energy/withdrawal subscales remained good or improved to excellent in most groups. The most notable change was for the children only group, where test-retest reliability of the pain subscale was moderate (ICC = 0.63) and the consolability subscale was good (ICC = 0.78) Please see additional file 6 for a full breakdown of test-retest reliability in these sub-groups.

#### Inter-rater reliability

Inter-rater reliability (*N* = 20) was completed by a second parent (*n* = 12, 60%), care or support worker (*n* = 4, 20%) or other family members (*n* = 2, 10%). Intraclass correlations showed that inter-rater reliability was moderate for the anxiety, pain and low energy/withdrawal subscales (ICC = 0.640, 0.690 and 0.712 respectively) and poor for the consolability subscale (ICC = 0.162).

### Validity

#### Convergent and divergent validity

To assess convergent and divergent validity of the measure, anxiety, pain and low energy/withdrawal subscale scores were correlated against scores on the generalised anxiety and depression subscales of the ADAMS and health questionnaire severity scores for painful items only. There was evidence for good convergent and divergent validity of the anxiety subscale, which correlated significantly with the ADAMS general anxiety subscale (*r* = .771, *p* < .001), but lower with the ADAMS depressed mood subscale (*r* = .469, *p* < .001), and health questionnaire severity scores (*r* = .230, *p* < .001).

Evidence for convergent and divergent validity of the pain subscale was mixed. Whilst the pain subscale correlated significantly against health questionnaire severity scores (*r* = .363, *p* < .001), it correlated more strongly with the ADAMS general anxiety and depressed mood subscales ([*r* = .522, *p* < .001], *r* = .413, *p* < .001]). However, of the ClASP-ID subscales, the pain subscale had the highest correlation with the health questionnaire severity scores.

There was evidence for good convergent and divergent validity of the low energy/withdrawal scale, which correlated most strongly with the ADAMS depressed mood subscale (*r* = .702, *p* < .001), and less strongly with the ADAMS general anxiety subscale and health questionnaire severity scores ([*r* = .476, *p* < .001], [*r* = .352, *p* < .001]). Please see Table 3 for a summary of these results.

**Table 3:** Convergent and divergent validity of the ClASP-ID subscales, Spearman’s rho correlations

	ADAMS General Anxiety	ADAMS Depressed Mood	Health Questionnaire Severity (painful only)
ClASP-ID Anxiety	0.771	0.469	0.230
ClASP-ID Pain	0.552	0.413	0.363
ClASP-ID low energy/withdrawal	0.476	0.702	0.352

Note: Due to missing data on some measures, N varied. Minimum *N* = 290

#### Known-groups validity

The discriminant validity of the ClASP-ID was explored by conducting t-tests to compare scores of clinical and non-clinical groups. Those whose parents or caregivers reported a clinical diagnosis of an anxiety disorder (*n* = 64) scored significantly higher on the anxiety subscale of the ClASP-ID than those without a reported clinical diagnosis of anxiety (*n* = 244) (t(306) = 6.02, *p* < .001). The effect size was large (*d* = 0.845). Individuals whose parents and caregivers reported a clinical diagnosis of depression (*n* = 20) scored significantly higher on the low energy/withdrawal scale of the ClASP-ID than those who did not report a diagnosis of depression (*n* = 289), (t(307) = 4.31, *p* < .001). The effect size was large (*d* = 0.846).

#### Known-groups validity: autistic group

To ensure the ClASP-ID was appropriate for autistic people specifically, further analysis was conducted. Autistic people with an anxiety disorder (ASD + anx, *n* = 49) scored significantly higher on the ClASP-ID anxiety subscale than those without an anxiety disorder diagnosis (ASD-anx, *n* = 107) by t-test (t(154) = 2.96, *p* = .004). There were no significant differences in SCQ scores between the ASD + anx group and the ASD-anx group (p_s_ > 0.05). Autistic people with a depression diagnosis (ASD + dep, *n* = 13) scored significantly higher on the ClASP-ID low energy/withdrawal subscale than those without a depression diagnosis (ASD-dep, *n* = 143) by t-test (t(153) = 4.69, *p* < .001). There were no significant differences in scores on the SCQ between the ASD + dep group and the ASD-dep group by (p_s_ > 0.05).

## Discussion

This study investigated the psychometric properties of the ClASP-ID in a large, heterogenous sample of people with intellectual disabilities. The ClASP-ID is one of few measures designed to screen for anxiety in people with severe intellectual disabilities, and it is suitable for people who speak few to no words and those with a diagnosis of autism. In this initial sample, the EFA revealed four factors that were labelled anxiety, pain, low energy/withdrawal and consolability. Low energy/withdrawal is a subscale that may be capturing depression symptomatology, but it was not labelled as a low/mood or depression scale given that mood related items did not load to the scale. Including items to screen for pain and low energy/withdrawal within the ClASP-ID prompts clinicians to consider differential diagnosis early within the assessment process, which is important due to the high level of overlap between behavioural indicators of anxiety, pain, and low mood in this group and the different intervention strategies required for each [22, 23, 26].

When exploring the results of the factor analysis and labelling latent variables, we considered the 14 items loading onto the largest factor to represent anxious behaviours (e.g. startle/appear on edge). Whilst we have labelled this factor ‘anxiety’, without assessment of cognition underlying the behaviours or whether behaviours occur in anticipation of a threat, rather than in direct response to a threat, it is possible the items are also indicative of a similar (albeit related) construct (e.g., fear). However, the ClASP-ID is not intended as a diagnostic measure of anxiety. Instead, it is our hope that the ClASP-ID can be used as a screening tool to prompt clinicians to consider anxiety, potential differential diagnoses and identify concerning behaviours requiring more in-depth diagnostic assessment, which may include observations to confirm antecedents of behaviours endorsed in the scale.

The psychometric properties of the ClASP-ID are promising, with the anxiety, pain, and low energy/withdrawal subscales showing excellent or good internal consistency, good test-retest reliability, and moderate inter-rater reliability. The consolability subscale, however, had lower internal consistency and inter-rater reliability, and moderate test-retest reliability, so caution is recommended until further testing of the subscale is conducted. There was evidence for good convergent and divergent validity of the ClASP-ID anxiety and low energy/withdrawal subscales. There also was encouraging evidence for the validity of the pain scale, although, due to mixed findings, further studies are important to validate this scale. Finally, there were significant differences between clinical groups and non-clinical groups at scale level. The effect size for this was large. Owing to statistical power, factor structure could not be examined in specific groups in the study. However, psychometric properties remained stable across a range of groups, including children, adults, autism, and those with the highest support needs.

The lower performance of the consolability subscale may reflect difficulties with assessing this construct. More variation could be expected in measures of consolability due to individual differences both within individuals and between raters. This scale asks questions that examine whether preparation before an event reduces distress and whether a person can be comforted. Preparation before an event may help an individual, but once they become distressed it may become difficult to provide comfort; or preparation before an event may not be sufficient to reduce distress, which only occurs if a person is removed from a situation. Hence variation across individuals and contexts may contribute to poor internal consistency scores. Further, consolability is expected to differ between two raters. For example, a primary caregiver may be able to calm or comfort an individual when distressed, whereas a second rater might have more difficulty.

When compared to the other psychometric properties, inter-rater reliability of the ClASP-ID subscales was lower. This is consistent with other measures reporting on people with intellectual disabilities, owing to the heterogeneity in behaviours shown across different contexts in this population and raters basing their assessment on behaviours across differing settings (for example, ADAMS; 34). Our second raters were primarily a second parent (*n* = 12, 60%), but also included care or support workers (*n* = 4, 20%) and extended family members (*n* = 2, 10%).

There are several possible explanations for the mixed pattern of results observed regarding the convergent and divergent validity of the pain subscale. Whilst the pain subscale correlated more strongly than any other ClASP-ID subscale with health questionnaire severity scores, it correlated most strongly with the ADAMS general anxiety subscale, followed by the ADAMS depressed mood subscale. These mixed results may be caused by the overlap in behavioural presentation between anxiety and pain in people who speak few or no words, making the context these behaviours occur in more important [24, 25]. It is also important to note that the ADAMS general anxiety subscale contains items such as “trembles when frightening situations are not present” which may be assessing an indicator of pain, hence strengthening the association between the ClASP-ID pain subscale and the ADAMS general anxiety subscale. Similarly, a small number of items such as “tearful” on the ADAMS depressed mood subscale may be capturing indicators of pain.

Further, anxiety and pain often co-occur in people with complex needs. For example, there is a link between anxiety and gastrointestinal (GI) difficulties in autistic people, which may interact with one another, causing a cycle of heightened anxiety and heightened GI difficulties [24, 63–65]. Despite this, the pain subscale yielded promising results and therefore warrants further evaluation using more direct assessments. For example, completing this scale pre and post intervention for physical health difficulties would indicate whether the scale captures change due to a reduction in pain, and if this was demonstrated this would provide insightful validation data.

One of the significant strengths of the ClASP-ID is that it was developed using a combination of approaches, including bottom-up interview methodology, literature reviews and examination of existing measures with the involvement of mental health clinicians. All items can be scored based on observations of a person’s behaviour and are carefully operationalised; hence completion of the ClASP-ID does not require family members to access the internal thoughts or feelings of the person they support. The initial item pool was comprehensive and included items that were representative of traditional anxiety symptomology and atypical anxiety indicators (e.g. repetitive behaviours) that have been highlighted as important anxiety markers in autistic individuals [30–32].

It is of interest that in the final version of the measure, repetitive behaviours were no longer included as they did not load on to the anxiety factor. One explanation for this may be that while repetitive behaviours are strongly associated with anxiety and are of clinical importance for detecting anxiety, increases in these behaviours may reflect a response or coping mechanism to reduce anxiety rather than being a core symptom of anxiety. In the same way that behaviours that challenge are often a response to anxiety but are not a behavioural equivalent of anxiety, this may also be true for repetitive behaviours [22]. Furthermore, repetitive behaviour may be an important indicator of anxiety for a subset of individuals but not all, hence reducing the likelihood of these behaviours loading on to the anxiety factor across a heterogeneous group of individuals with severe intellectual disabilities.

The present study had some limitations that will need to be addressed in further studies. First, during interview development it was not feasible to conduct cognitive interviews to establish that parents and caregivers interpreted the questions correctly due to the number of items included in the initial version of the measure and the available timeframe for completing the study. Further, development did not include autistic people with intellectual disabilities themselves. A future study could evaluate the face-validity and acceptability of the measure for autistic people and people with intellectual disabilities, however, it is also important to recognise that the behavioural presentation of anxiety of people with mild intellectual disabilities may differ considerably from those with severe intellectual disabilities. So, whilst such a study would provide insight into the views of this population, the generalisability of these views to all individuals with intellectual disabilities could be a limitation of this approach.

Secondly, small sample sizes for the inter-rater reliability calculations (*n* = 20, 6% of sample) meant we were not able to use this to refine the ClASP-ID. Some items on the ClASP-ID have poor inter-rater reliability, and at present it is unclear if this is a consequence of a small sample size and high variability in relationship of the second rater to the person with intellectual disability, or poorly performing items. Further work should seek to investigate the item-level inter-rater reliability of the ClASP-ID in a larger sample. In addition, replicating this work in a large independent mixed aetiology group would allow the factor structure to be examined in larger samples of more specific groups (such as children only, adults only, or autistic groups only) where there was not adequate statistical power to do so in the present study. Finally, this larger sample would also allow for confirmatory factor analysis of this initial factor structure to be conducted.

This study was also reliant on parent reports of anxiety disorder diagnosis to define clinical groups and assess known-groups validity. Issues with anxiety diagnosis in people with severe to profound intellectual disabilities, such as diagnostic overshadowing and a lack of suitability of standard diagnostic criteria mean it is likely that the non-anxious group contains a substantial proportion of individuals with clinical levels of anxiety that is undiagnosed [20, 21, 66]. Known-groups validity of the measure may be strengthened once clinical groups are defined by direct assessment or physiological measures of anxiety as opposed to proxy report. Further work is ongoing to assess the validity of the ClASP-ID in samples where direct and clinical assessments of anxiety are being conducted.

Further work is needed to replicate the findings of this study across a range of intellectual abilities, using more in-depth measures of ability level which were not feasible to conduct in the present study due to the large sample size. As the ClASP-ID relies on behavioural markers of anxiety, we expect the measure may perform well in people with mild intellectual disabilities, but this has not yet been fully investigated owing to the smaller proportion of people with mild to moderate intellectual disabilities in this sample. In-depth assessments of ability level would provide additional confidence about the groups the ClASP-ID is most effective for and provide additional evidence of its utility for detecting anxiety in severe intellectual disabilities.

Whilst there is good evidence that the ClASP-ID is likely to be a measure that screens for anxiety across groups, it will be beneficial to validate the ClASP-ID in rare genetic syndromes associated with intellectual disabilities. Whilst rare genetic syndromes were included in the present study, recruitment was not focused on specific syndrome groups. Some syndromes may require additional validation given the complexity of anxiety, low mood, and pain presentations [32]. Similarly, whilst both adults and children with intellectual disabilities were included in this study, there were only a small proportion of adults in the study who lived away from their family (for example, in supported living). Data from adults not living with their family were included in the present study, although numbers were too low to conduct any psychometric analysis. Family members of adults with intellectual disability living away from home may have a different perspective on the person they care for, and future work should seek to validate the measure specifically in adults with intellectual disability not living with their parents.

## Conclusions

In summary, this study has demonstrated that the ClASP-ID is a promising tool for the measurement of anxiety in children and adults with severe intellectual disabilities. In particular, the tool addresses a significant gap in the literature because it is suitable for people who speak few to no words, who have a co-diagnosis of autism, and across a range of ages. By prompting clinicians and researchers to consider differential diagnosis, the tool facilitates precision in measurement and assessment. The tool has significant potential to enhance research in the neglected field of mental health in people with severe intellectual disabilities. Furthermore, detecting anxiety earlier in people with intellectual disabilities increases the likelihood of improving the long-term wellbeing and outcomes of individuals and their caregivers.

### Electronic supplementary material

Below is the link to the electronic supplementary material.

**Additional file 1**- List of measures consulted during ClASP-ID development.

**Additional file 2**- Interview study data.

**Additional file 3**- Beta version of the ClASP-ID and list of items removed to form the final ClASP-ID.

**Additional file 4**- Parent questionnaire feedback proforma.

**Additional file 5**- Demographic information for the inter-rater reliability and test-retest reliability samples.

**Additional file 6**- Further psychometric properties of the ClASP-ID, including internal consistency and test-retest reliability of specific sample subgroups.

## Data Availability

The datasets used and/or analysed during the current study are available from the corresponding author on reasonable request.

## Footnotes

1 Throughout this paper, we use identity first language to refer to autistic people, in line with guidelines from Autistica and current preferences from the autistic community (11–12).

## Abbreviations

ADAMS: Anxiety, Depression and Mood Scale
ADIS/ASA: Anxiety Disorders Interview Schedule-Autism Addendum
ASC-ASD: Anxiety Scale for Children with Autism Spectrum Disorder
ClASP-ID: Clinical Anxiety Scale for People with Intellectual Disabilities
DASH-II: Diagnostic Assessment for the Severely Handicapped, second edition
ID: Intellectual Disability
MIPQ: Mood Interest and Pleasure Questionnaire
SCQ: Social Communication Questionnaire

## References

[1] Emerson E, Hatton C. Mental health of children and adolescents with intellectual disabilities in Britain. Br J Psychiatry. 2007;191:493–9. 18055952 10.1192/bjp.bp.107.038729

[2] Merikangas KR, Nakamura EF, Kessler RC. Epidemiology of mental disorders in children and adolescents. Dialog Clin Neurosci. 2009;11(1):7–20. 10.31887/DCNS.2009.11.1/krmerikangas PMC2807642 19432384

[3] Singh S, Singh LK, Sahu M, Tikka SK. Do comorbidities among patients with mental retardation differ across various age groups? Asian J Psychiatry. 2019;39. 10.1016/j.ajp.2018.11.001 30466056

[4] White P, Chant D, Edwards N, Townsend C, Waghorn G. Prevalence of intellectual disability and comorbid mental illness in an Australian community sample. Aust N Z J Psychiatry. 2005;39(5):395–400. 15860028 10.1080/j.1440-1614.2005.01587.x

[5] Edwards G, Jones C, Pearson E, Royston R, Oliver C, Tarver J et al. Prevalence of anxiety symptomatology and diagnosis in syndromic intellectual disability: a systematic review and meta-analysis. Neurosci Biobehav Rev. 2022;138. 10.1016/j.neubiorev.2022.104719 35661754

[6] Groves L, Moss J, Oliver C, Royston R, Waite J, Crawford H. Divergent presentation of anxiety in high-risk groups within the intellectual disability population. J Neurodevelopmental Disorders. 2022;14(1). 10.1186/s11689-022-09462-w PMC9535841 36199025

[7] Wolstencroft J, Wicks F, Srinivasan R, Wynn S, Ford T, Baker K, et al. Neuropsychiatric risk in children with intellectual disability of genetic origin: IMAGINE, a UK national cohort study. Lancet Psychiatry. 2022;9(9):715–24. 35932790 10.1016/S2215-0366(22)00207-3 PMC9636306

[8] Arvio M, Sillanpaa M. Prevalence, aetiology and comorbidity of severe and profound intellectual disability in Finland. J Intellect Disabil Res. 2003;47:108–12. 12542576 10.1046/j.1365-2788.2003.00447.x

[9] Chadwick O, Piroth N, Walker J, Bernard S, Taylor E. Factors affecting the risk of behaviour problems in children with severe intellectual disability. J Intellect Disabil Res. 2000;44:108–23. 10898374 10.1046/j.1365-2788.2000.00255.x

[10] Patel DR, Apple R, Kanungo S, Akkal A. Narrative review of intellectual disability: definitions, evaluation and principles of treatment. Pediatr Med. 2018;1:11. 10.21037/pm.2018.12.02

[11] Autistica. Media communications guide https://www.autistica.org.uk/about-us/media-communications-guide2019.

[12] Kenny L, Hattersley C, Molins B, Buckley C, Povey C, Pellicano E. Which terms should be used to describe autism? Perspectives from the UK autism community. Autism. 2016;20(4):442–62. 26134030 10.1177/1362361315588200

[13] Buckley N, Glasson EJ, Chen W, Epstein A, Leonard H, Skoss R, et al. Prevalence estimates of mental health problems in children and adolescents with intellectual disability: a systematic review and meta-analysis. Aust N Z J Psychiatry. 2020;54(10):970–84. 32475125 10.1177/0004867420924101

[14] Hollocks MJ, Lerh JW, Magiati I, Meiser-Stedman R, Brugha TS. Anxiety and depression in adults with autism spectrum disorder: a systematic review and meta-analysis. Psychol Med. 2019;49(4):559–72. 30178724 10.1017/S0033291718002283

[15] Tonnsen BL, Boan AD, Bradley CC, Charles J, Cohen A, Carpenter LA. Prevalence of Autism Spectrum disorders among Children with Intellectual disability. Ajidd-American J Intellect Dev Disabil. 2016;121(6):487–500. 10.1352/1944-7558-121.6.487 27802102

[16] Alvaro PK, Roberts RM, Harris JK. A systematic review assessing bidirectionality between sleep disturbances, anxiety, and Depression. Sleep. 2013;36(7):1059–68. 23814343 10.5665/sleep.2810 PMC3669059

[17] Bishop C, Mulraney M, Rinehart N, Sciberras E. An examination of the association between anxiety and social functioning in youth with ADHD: a systematic review. Psychiatry Res. 2019;273:402–21. 30684786 10.1016/j.psychres.2019.01.039

[18] Kerns CM, Kendall PC, Zickgraf H, Franklin ME, Miller J, Herrington J. Not to be overshadowed or overlooked: functional impairments Associated with Comorbid anxiety disorders in Youth with ASD. Behav Ther. 2015;46(1). 10.1016/j.beth.2014.03.005 25526833

[19] van Steensel FJA, Bogels SM, Dirksen CD. Anxiety and quality of life: clinically anxious children with and without Autism Spectrum disorders compared. J Clin Child Adolesc Psychol. 2012;41(6):731–8. 22775580 10.1080/15374416.2012.698725

[20] Flynn S, Vereenooghe L, Hastings RP, Adams D, Cooper SA, Gore N, et al. Measurement tools for mental health problems and mental well-being in people with severe or profound intellectual disabilities: a systematic review. Clin Psychol Rev. 2017;57:32–44. 28821007 10.1016/j.cpr.2017.08.006

[21] Bailey NM, Andrews TM. Diagnostic Criteria for Psychiatric disorders for Use with adults with Learning Disabilities/Mental Retardation (DC-LD) and the diagnosis of anxiety disorders: a review. J Intellect Disabil Res. 2003;47:50–61. 14516374 10.1046/j.1365-2788.47.s1.25.x

[22] Eaton C, Tarver J, Shirazi A, Pearson E, Walker L, Bird M, et al. A systematic review of the behaviours associated with depression in people with severe-profound intellectual disability. J Intellect Disabil Res. 2021;65(3):211–29. 33426741 10.1111/jir.12807

[23] Merkel SI, Voepel-Lewis T, Shayevitz JR, Malviya S. The FLACC: a behavioral scale for scoring postoperative pain in young children. Pediatr Nurs. 1997;23(3):293–7. 9220806

[24] Oliver C, Adams D, Allen D, Crawford H, Heald M, Moss J, et al. The behaviour and wellbeing of children and adults with severe intellectual disability and complex needs: the Be-well checklist for carers and professionals. Paediatrics Child Health. 2020;30(12):416–24. 10.1016/j.paed.2020.09.003

[25] Tarver J, Pearson E, Edwards G, Shirazi A, Potter L, Malhi P, et al. Anxiety in autistic individuals who speak few or no words: a qualitative study of parental experience and anxiety management. Autism. 2021;25(2):429–39. 32998530 10.1177/1362361320962366 PMC7874371

[26] Helverschou SB, Martinsen H. Anxiety in people diagnosed with autism and intellectual disability: Recognition and phenomenology. Res Autism Spectr Disorders. 2011;5(1):377–87. 10.1016/j.rasd.2010.05.003

[27] Breau LM, Camfield CS, McGrath PJ, Finley GA. The incidence of Pain in Children with severe cognitive impairments. Arch Pediatr Adolesc Med. 2003;157(12):1219–26. 14662579 10.1001/archpedi.157.12.1219

[28] Liao P, Vajdic C, Trollor J, Reppermund S. Prevalence and incidence of physical health conditions in people with intellectual disability – a systematic review. PLoS ONE. 2021;16(8):e0256294. 34428249 10.1371/journal.pone.0256294 PMC8384165

[29] Oliver C, Ellis K, Agar G, Bissell S, Chung JCY, Crawford H, et al. Chapter four - distress and challenging behavior in people with profound or severe intellectual disability and complex needs: Assessment of causes and evaluation of intervention outcomes. In: Esbensen AJ, Schworer EK, editors. International Review of Research in Developmental Disabilities. Volume 62. Academic; 2022. pp. 109–89.

[30] Baribeau DA, Vigod S, Pullenayegum E, Kerns CM, Mirenda P, Smith IM, et al. Repetitive behavior severity as an early Indicator of risk for elevated anxiety symptoms in Autism Spectrum Disorder. J Am Acad Child Adolesc Psychiatry. 2020;59(7):890–9. 31541676 10.1016/j.jaac.2019.08.478

[31] Rodgers J, Glod M, Connolly B, McConachie H. The relationship between anxiety and repetitive behaviours in Autism Spectrum Disorder. J Autism Dev Disord. 2012;42(11):2404–9. 22527704 10.1007/s10803-012-1531-y

[32] Crawford H, Oliver C, Groves L, Bradley L, Smith K, Hogan A, et al. Behavioural and physiological indicators of anxiety reflect shared and distinct profiles across individuals with neurogenetic syndromes. Psychiatry Res. 2023;326:115278. 37285621 10.1016/j.psychres.2023.115278

[33] Hallett V, Lecavalier L, Sukhodolsky DG, Cipriano N, Aman MG, McCracken JT, et al. Exploring the manifestations of anxiety in children with Autism Spectrum disorders. J Autism Dev Disord. 2013;43(10):2341–52. 23400347 10.1007/s10803-013-1775-1 PMC4038127

[34] Esbensen AJ, Rojahn J, Aman MG, Ruedrich S. Reliability and validity of an assessment instrument for anxiety, depression, and mood among individuals with mental retardation. J Autism Dev Disord. 2003;33(6):617–29. 14714931 10.1023/B:JADD.0000005999.27178.55

[35] American Psychiatric Association. Diagnostic and statistical manual of mental disorders (3 ed. rev.)1987.

[36] Marston GM, Perry DW, Roy A. Manifestations of depression in people with intellectual disability. J Intellect Disabil Res. 1997;41:476–80. 9430051 10.1111/j.1365-2788.1997.tb00739.x

[37] Appleton H, Roberts J, Simpson K. How is anxiety identified and diagnosed in individuals with Autism Spectrum disorder and intellectual disability? A scoping review. J Mental Health Res Intellect Disabil. 2019;12(3–4):152–75. 10.1080/19315864.2019.1679299

[38] Edwards G, Tarver J, Shelley L, Bird M, Hughes J, Crawford H, et al. Utilising interview methodology to inform the development of New Clinical Assessment Tools for anxiety in autistic individuals who speak few or no words. J Autism Dev Disord. 2023;53(6):2328–48. 35304663 10.1007/s10803-022-05509-y PMC10229722

[39] Rodgers J, Wigham S, McConachie H, Freeston M, Honey E, Parr JR. Development of the anxiety scale for children with autism spectrum disorder (ASC-ASD). Autism Res. 2016;9(11):1205–15. 26887910 10.1002/aur.1603

[40] Kerns CM, Renno P, Kendall PC, Wood JJ, Storch EA. Anxiety disorders interview schedule-autism addendum: reliability and validity in Children with Autism Spectrum Disorder. J Clin Child Adolesc Psychol. 2017;46(1):88–100. 27925775 10.1080/15374416.2016.1233501 PMC5441235

[41] Pitt HL. Behavioural Markers of Anxiety and Depression in Individuals with Intellectual Disabilities [Unpublished Masters Dissertation.]: University of Birmingham; 2018.

[42] Eden KE. The association between pain and challenging behaviour in people with intellectual disability [Doctoral dissertation]: University of Birmingham; 2013.

[43] de Knegt NC, Pieper MJC, Lobbezoo F, Schuengel C, Evenhuis HM, Passchier J, et al. Behavioral Pain indicators in people with Intellectual disabilities: a systematic review. J Pain. 2013;14(9):885–96. 23830762 10.1016/j.jpain.2013.04.016

[44] McConachie H, Parr JR, Glod M, Hanratty J, Livingstone N, Oono IP, et al. Systematic review of tools to measure outcomes for young children with autism spectrum disorder. Health Technol Assess. 2015;19(41):1–506. 26065374 10.3310/hta19410 PMC4781156

[45] Royston R, Oliver C, Howlin P, Waite J. Anxiety characteristics in individuals with Williams syndrome. J Appl Res Intellect Disabil. 2021;34(4):1098–107. 33561900 10.1111/jar.12864

[46] Finstad K. Response interpolation and scale sensitivity: evidence against 5-point scales. J Usability Stud. 2010;5(3):104–10.

[47] Lietz P. Research into Questionnaire Design: a Summary of the literature. Int J Market Res. 2010;52(2):249–72. 10.2501/S147078530920120X

[48] Oaster TRF. Number of Alternatives per Choice Point and Stability of Likert-Type scales. Percept Mot Skills. 1989;68(2):549–50. 10.2466/pms.1989.68.2.549

[49] Fife-Schaw C. Questionnaire Development. Research methods in psychology. 5 ed. London, UK: SAGE publications ltd; 2020.

[50] Kushlick A, Blunden R, Cox G. Method of rating behavior characteristics for use in large-scale surveys of mental handicap. Psychol Med. 1973;3(4):466–78. 4762223 10.1017/S0033291700054271

[51] Carulla LS, Reed GM, Vaez-Azizi LM, Cooper SA, Leal RM, Bertelli M, et al. Intellectual developmental disorders: towards a new name, definition and framework for mental retardation/intellectual disability in ICD‐11. World Psychiatry. 2011;10(3):175–80. 21991267 10.1002/j.2051-5545.2011.tb00045.x PMC3188762

[52] Leclezio L, Jansen A, Whittemore VH, de Vries PJ. Pilot validation of the Tuberous Sclerosis-Associated Neuropsychiatric disorders (TAND) checklist. Pediatr Neurol. 2015;52(1):16–24. 25499093 10.1016/j.pediatrneurol.2014.10.006

[53] Moss J, Richards C, Nelson L, Oliver C. Prevalence of autism spectrum disorder symptomatology and related behavioural characteristics in individuals with Down syndrome. Autism. 2013;17(4):390–404. 22589453 10.1177/1362361312442790

[54] Waite J, Moss J, Beck SR, Richards C, Nelson L, Arron K, et al. Repetitive behavior in Rubinstein-Taybi Syndrome: parallels with Autism Spectrum Phenomenology. J Autism Dev Disord. 2015;45(5):1238–53. 25491025 10.1007/s10803-014-2283-7

[55] Hall SS, Arron K, Sloneem J, Oliver C. Health and sleep problems in Cornelia De Lange Syndrome: a case control study. J Intellect Disabil Res. 2008;52:458–68. 18341525 10.1111/j.1365-2788.2008.01047.x

[56] Brooks WT, Benson BA. The validity of the social communication questionnaire in adults with intellectual disability. Res Autism Spectr Disorders. 2013;7(2):247–55. 10.1016/j.rasd.2012.10.002

[57] Chesnut SR, Wei TL, Barnard-Brak L, Richman DM. A meta-analysis of the social communication questionnaire: screening for autism spectrum disorder. Autism. 2017;21(8):920–8. 27503464 10.1177/1362361316660065

[58] Mokkink LB, de Vet HCW, Prinsen CAC, Patrick DL, Alonso J, Bouter LM, et al. COSMIN Risk of Bias checklist for systematic reviews of patient-reported outcome measures. Qual Life Res. 2018;27(5):1171–9. 29260445 10.1007/s11136-017-1765-4 PMC5891552

[59] Prinsen CAC, Mokkink LB, Bouter LM, Alonso J, Patrick DL, de Vet HCW, et al. COSMIN guideline for systematic reviews of patient-reported outcome measures. Qual Life Res. 2018;27(5):1147–57. 29435801 10.1007/s11136-018-1798-3 PMC5891568

[60] Terwee CB, Prinsen CAC, Chiarotto A, Westerman MJ, Patrick DL, Alonso J, et al. COSMIN methodology for evaluating the content validity of patient-reported outcome measures: a Delphi study. Qual Life Res. 2018;27(5):1159–70. 29550964 10.1007/s11136-018-1829-0 PMC5891557

[61] Williams B, Onsman A, Brown T. Exploratory factor analysis: a five-step guide for novices. Australasian J Paramedicine. 2010;8:1–13. 10.33151/ajp.8.3.93

[62] Beavers A, Lounsbury J, Richards J, Schuyler H, Skolits G, Esquivel S. Practical Considerations for Using Exploratory Factor Analysis in Educational Research. Practical Assessment, Research, and Evaluation. 2019;18(6).

[63] Mazurek MO, Vasa RA, Kalb LG, Kanne SM, Rosenberg D, Keefer A, et al. Anxiety, sensory over-responsivity, and gastrointestinal problems in children with Autism Spectrum disorders. J Abnorm Child Psychol. 2013;41(1):165–76. 22850932 10.1007/s10802-012-9668-x

[64] Wasilewska J, Klukowski M. Gastrointestinal symptoms and autism spectrum disorder: links and risks - a possible new overlap syndrome. Pediatr Health Med Ther. 2015;6:153–66. 10.2147/PHMT.S85717 PMC5683266 29388597

[65] William S, Leader G, Mannion A, Chen J. An investigation of anxiety in children and adolescents with autism spectrum disorder. Res Autism Spectr Disorders. 2015;10:30–40. 10.1016/j.rasd.2014.10.017

[66] Winder-Patel B, Tudor ME, Kerns CM, Davis K, Nordahl CW, Amaral DG, et al. Often undiagnosed but Treatable: case vignettes and clinical considerations for assessing anxiety disorders in Youth with Autism Spectrum disorder and intellectual disability. Evidence-based Pract Child Adolesc Mental Health. 2022;7(1):24–40. 10.1080/23794925.2021.1923090 PMC8916744 35284637

[67] Matson JL, Rush KS, Hamilton M, Anderson SJ, Bamburg JW, Baglio CS, et al. Characteristics of depression as assessed by the Diagnostic Assessment for the severely Handicapped-II (DASH-II). Res Dev Disabil. 1999;20(4):305–13. 10425658 10.1016/S0891-4222(99)00012-8

[68] Rutter M, Bailey A, Lord C. SCQ. The Social Communication Questionnaire. Torrance, CA.: Western Psychological Services; 2003.

