# Supplementary material for: Development and psychometric properties of the Clinical Anxiety Scale for People with Intellectual Disabilities (ClASP-ID)
Source: J Neurodev Disord. 2024 Jul 27;16:43. doi: 10.1186/s11689-024-09554-9 (PMC11283710; doi:10.1186/s11689-024-09554-9)
Supplement: Supplementary file 1 — Additional file 1- List of measures consulted during ClASP-ID development. [file 11689_2024_9554_MOESM1_ESM.docx]

**Additional File 1- Measures Consulted During ClASP-ID Development**

| Acronym | Measure | | Authors |
| --- | --- | --- | --- |
| AADS | | Assessment for Adults with Developmental Disabilities | McQuillan, Kalsy, Oliver & Hall (2001) |
| ABC | | The Aberrant Behaviour Checklist | Aman and Singh (1985) |
| ABBEY | | Abbey Pain Scale | Abbey et al. (2004) |
| ADAMS | | The Anxiety, Depression and Mood Scale | Esbensen et al. (2003) |
| ASD-ASC | | Anxiety Scale for Children- Autism Spectrum Disorder | Rodgers et al., (2016) |
| ASD-CA | | Autistic Spectrum Disorder - Comorbidity for Adults | Matson and Boisjoli (2008) |
| BAI | | Beck Anxiety Inventory | Beck and Steer (1990) |
| BAPS | | Bolton Pain Assessment Scale | Royal Bolton Hospital NHS Foundation Trust (2011) |
| BASC-2 | | Behavioural Assessment System for Children-2 | Reynolds & Kamphaus (2004) |
| BDI | | Beck Depression Inventory | Beck et al. (1961) |
| BDSRS | | Birleson Depressive Short Form Self-Rating Scale | Birleson (1981); Hartley and McLean (2009) |
| CBCL | | The Child Behaviour Checklist | Achenbach & Rescorla (2001) |
| CNPI | | Checklist of Non-Verbal Pain Indicators | Feldt (2000) |
| CPS-NAID | | The Chronic Pain Scale for Non-Verbal Adults with Intellectual Disabilities | Breau et al., (2009) |
| DASH | | Diagnostic Assessment for the Severly Handicapped | Matson et al.(1991) |
| DASH-II | | Diagnostic Assessment for the Severly Handicapped - II | Matson (1995) |
| DBC-T | | Developmental Behaviour Checklist - Teacher Version | Einfeld and Tonge (1989) |
| DEPRESSED | | Depression Scale for Severe Disability | Cooper (2007) |
| GAS | | The Glasgow Anxiety Scale | Mindham and Espie (2003) |
| GDS-CS | | The Glasgow Depression Scale - Carer Supplement | Cuthill et al. (2003) |
| GDS-LD | | The Glasgow Depression Scale - Learning Disability | Cuthill et al. (2003) |
| HADS | | Hospital Anxiety and Depression Scale | Dagnan et al. (2008); Zigmond and Snaith (1983) |
| MIPQ | | Mood, Interest and Pleasure Questionnaire | Ross & Oliver (2002, 2003) |
| NCAPC | | The Non-Communicating Adult Pain Checklist | Lotan et al. (2009) |
| NCCPC-PV | | The Non-Communicating Children's Pain Checklist - Postperative Version | Breau et al. (2002) |
| r-FLACC | | Revised-Face, Legs, Activity, Cry, Consolability | Voepel-Lewis et al. (2008) |
| RSMB | | The Reiss Screen for Maladaptive Behaviour | Reiss (1988) |
| SAS | | The Zung Self-Rating Anxiety Scale | Zung (1971) |
| SCARED-C | | Screening for Childhood Anxiety and Related Emotional Disorders - Child Verson | Birmharer et al.(1997; 1999) |
| SCARED-P | | Screening for Childhood Anxiety and Related Emotional Disorders - Parent Version | Birmharer et al.(1997; 1999) |
| SCAS | | The Spence Anxiety Scales | Spence (1997; 1999) |
| SCAS-P | | Spence Children's Anxiety Rating Scale - Parent Report | Spence (1997; 1999) |
| SCL-90-R | | Symptom Checklist 90-R | Derogatis (1983); Kellett et al. (1999) |
| SDS | | The Zung Self-Rating Depression Scale | Zung (1965) |
| SRDQ | | Self-Report Depression Questionnaire | Esbensen & Benson (2007); Esbensen et al.(2005) |
| STAIC | | The State Trait Anxiety Inventory for Children | Spielberger (1973) |
