# Supplementary material for: Development and psychometric properties of the Clinical Anxiety Scale for People with Intellectual Disabilities (ClASP-ID)
Source: J Neurodev Disord. 2024 Jul 27;16:43. doi: 10.1186/s11689-024-09554-9 (PMC11283710; doi:10.1186/s11689-024-09554-9)
Supplement: Supplementary file 2 — Additional file 2- Interview study data. [file 11689_2024_9554_MOESM2_ESM.docx]

| *Characteristics of sample for interview study (n=30).* | | |
| --- | --- | --- |
|  | Mean (SD) | Range |
| Age of parent/carer (years) | 48.9 (7.1) | 37-66 |
| Age of person with ID (years) | 16.7 (10.6) | 4-52 |
| SCQ total score | 23.3 (6.5) | 2-31 |
| ADAMS generalised anxiety score | 10.8 (4.5) | 2-20 |
| ADAMS social avoidance score | 9.9 (4.7) | 2-20 |
|  | *n* (%) |  |
| Person with ID male | 22 (73.3) |  |
| Parent/carer female | 27 (90) |  |
| Wessex Self-help score |  |  |
| Not able | 7 (23.3) |  |
| Partly able | 21 (70) |  |
| Able | 2 (6.7) |  |
| Wessex verbal ability |  |  |
| Non-verbal | 7 (23.3) |  |
| Odd words only | 23 (76.7) |  |
| Genetic syndrome diagnosis |  |  |
| Cornelia de Lange syndrome | 1 (3.3) |  |
| Fragile X Syndrome | 1 (3.3) |  |
| Soto syndrome | 1 (3.3) |  |
| 9q34 deletion | 1 (3.3) |  |
| Tuberous Sclerosis | 2 (6.7) |  |
| Kleefstra Syndrome | 3 (10) |  |
| Down Syndrome | 1 (3.3) |  |
| Autism | 24 (80) |  |
| Note SCQ= Social Communication Questionnaire; ADAMS= Anxiety, Depression and Mood Scale | | |

**Additional File 2- Interview Study Data**

| *Frequencies of coded anxiety triggers.* | |
| --- | --- |
|  | *n* (%) |
| Social interactions/performance situations/high demands | 14 (46.7) |
| Separation/being alone | 3 (10) |
| Everyday events/multiple contexts or situations | 3 (10) |
| Routine changes | 20 (66.7) |
| New situations/unfamiliar settings | 14 (46.7) |
| Worry about future events | 4 (13.3) |
| Lack of sleep | 3 (10) |
| Sensory overload | 18 (60) |
| Aversive setting (e.g., school) | 7 (23.3) |
| Illness | 6 (20) |
| Specific phobia | 13 (43.3) |

| *Frequencies of behaviours associated with anxiety.* | |
| --- | --- |
|  | *n* (%) |
| Blushing | 4 (13.3) |
| Trembling/Shaking | 5 (16.7) |
| Palpitations/pound heart | 2 (6.7) |
| Difficulty breathing | 6 (20) |
| Self-injury | 16 (53.3) |
| Aggression towards others | 14 (46.7) |
| Crying | 9 (30) |
| Repetitive behaviours | 14 (46.7) |
| Need to flee/keep distance | 13 (43.3) |
| Tense face/furrowed brow | 8 (26.7) |
| Grimacing | 2 (6.7) |
| Frowning | 3 (10) |
| Talking less/decreased vocalisation | 4 (13.3) |
| Increased vocalisation/shouting | 22 (73.3) |
| Talks to others | 1 (3.3) |
| Repetitive speech | 13 (43.3) |
| Sweating | 3 (10) |
| Nausea/feeling sick | 4 (13.3) |
| Increased urge to go to the bathroom | 3 (10) |
| Jumpy/on edge | 2 (6.7) |
| Refusal to listen/cooperate | 4 (13.3) |
| Decreased activity or movement | 8 (26.7) |
| Pacing/restlessness/increased movement | 13 (43.3) |
| Self-soothing behaviour | 4 (13.3) |
| Throws objects /slams doors/breaks things | 10 (33.3) |
| Muscle Tension | 12 (40) |
| Decreased movement | 4 (13.3) |
| Mumbling | 1 (3.3) |
| Tone change | 5 (16.7) |
