# Supplementary material for: Development and psychometric properties of the Clinical Anxiety Scale for People with Intellectual Disabilities (ClASP-ID)
Source: J Neurodev Disord. 2024 Jul 27;16:43. doi: 10.1186/s11689-024-09554-9 (PMC11283710; doi:10.1186/s11689-024-09554-9)
Supplement: Supplementary file 3 — Additional file 3- Beta version of the ClASP-ID and list of items removed to form the final ClASP-ID. [file 11689_2024_9554_MOESM3_ESM.docx]

**Additional File 3- Beta version of the ClASP-ID**

*Beta version of the ClASP-ID*

**Clinical Anxiety Screen for people with Severe to Profound Intellectual Disability (ClASP-ID)**

 This questionnaire is going to ask you about behaviours you may or may not have seen in the person you care for, over the last MONTH.
For each item, you will be asked to rate how frequently the behaviours have occurred over the last month.
For some of the questions, we will also ask you to think about whether the behaviour has been occurring more or less than is usual for the person you care for, over the last month.
Please try to answer every question.
If you are unsure whether you have seen the behaviour, please select ‘almost never’ and move onto the next question

| **Has he/she…** |  |
| --- | --- |
| 1. **Been clinging to or seeking physical comfort from someone familiar?** | **1b. Over the past one month, has this behaviour been…** |
| Almost never Once a month Less than Once or twice At least 3-4 times Every day More than  once a week a week per week once a day | Occurring a lot more than is typical of him/her |
|  | Occurring a bit more than is typical of him/her |
|  | Occurring at the same rate that is typical of him/her |
|  | Occurring a bit less than is typical of him/her |
|  | Occurring a lot less than is typical of him/her |
| **Has he/she…** |  |
| 1. **Been asking repetitive questions?** | **2b. Over the past one month, has this behaviour been…** |
| Almost never Once a month Less than Once or twice At least 3-4 times Every day More than  once a week a week per week once a day | Occurring a lot more than is typical of him/her |
|  | Occurring a bit more than is typical of him/her |
|  | Occurring at the same rate that is typical of him/her |
|  | Occurring a bit less than is typical of him/her |
|  | Occurring a lot less than is typical of him/her |
| **Does he/she…** |  |
| 1. **Appear quiet and/or less active?** | **3b. Over the past one month, has this behaviour been…** |
| Almost never Once a month Less than Once or twice At least 3-4 times Every day More than  once a week a week per week once a day | Occurring a lot more than is typical of him/her |
|  | Occurring a bit more than is typical of him/her |
|  | Occurring at the same rate that is typical of him/her |
|  | Occurring a bit less than is typical of him/her |
|  | Occurring a lot less than is typical of him/her |
| **Has he/she…** |  |
| 1. **Seemed withdrawn or ‘vacant’?** | **4b. Over the past one month, has this behaviour been…** |
| Almost never Less than a quarter Less than About half of More than half About three All of the  of the time half the time the time the time quarters of time  the time | Occurring a lot more than is typical of him/her |
|  | Occurring a bit more than is typical of him/her |
|  | Occurring at the same rate that is typical of him/her |
|  | Occurring a bit less than is typical of him/her |
|  | Occurring a lot less than is typical of him/her |
| **Does he/she…** |  |
| 1. **Throw himself/herself to the ground or thrash around?** |  |
| Almost never Once a month Less than Once or twice At least 3-4 times Every day More than  once a week a week per week once a day | |
|  | |
| 1. **ever make negative or frustrated vocalisations? (e.g. whining, grumbling, growling, shouting, screaming)** |  |
| Almost never Once a month Less than Once or twice At least 3-4 times Every day More than  once a week a week per week once a day | |
|  | |
| 1. **appear on edge OR on the look out for danger?** |  |
| Almost never Once a month Less than Once or twice At least 3-4 times Every day More than  once a week a week per week once a day | |
|  | |
| 1. **ever become tense, stiff or rigid?** |  |
| Almost never Once a month Less than Once or twice At least 3-4 times Every day More than  once a week a week per week once a day | |
|  | |
| 1. **ever appear more floppy than usual?** |  |
| Almost never Once a month Less than Once or twice At least 3-4 times Every day More than  once a week a week per week once a day | |
|  | |
| 1. **ever seem protective of a particular part of his/her body? (e.g. holding it, guarding it, flinching)** |  |
| Almost never Once a month Less than Once or twice At least 3-4 times Every day More than  once a week a week per week once a day | |
|  | |
| **Does he/she…** |  |
| 1. **pace around the room?** |  |
| Almost never Once a month Less than Once or twice At least 3-4 times Every day More than  once a week a week per week once a day | |
|  | |
| 1. **ever appear restless or agitated?** |  |
| Almost never Once a month Less than Once or twice At least 3-4 times Every day More than  once a week a week per week once a day | |
|  | |
| 1. **ever run away or hide from certain objects or situations?** |  |
| Almost never Once a month Less than Once or twice At least 3-4 times Every day More than  once a week a week per week once a day | |
|  | |
| 1. **ever cover himself/herself with a blanket or try to place a barrier between himself/herself and others or a situation?** |  |
| Almost never Once a month Less than Once or twice At least 3-4 times Every day More than  once a week a week per week once a day | |
|  | |
| 1. **cry or well up as if he/she is about to cry?** |  |
| Almost never Once a month Less than Once or twice At least 3-4 times Every day More than  once a week a week per week once a day | |
|  | |
| 1. **ever have watery eyes that is different from crying?** |  |
| Almost never Once a month Less than Once or twice At least 3-4 times Every day More than  once a week a week per week once a day | |
|  | |
| **Does he/she…** |  |
| 1. **ever freeze suddenly (stick to the spot) in response to specific situations?** |  |
| Almost never Once a month Less than Once or twice At least 3-4 times Every day More than  once a week a week per week once a day | |
|  | |
| 1. **ever cover his/her eyes or ears?** |  |
| Almost never Once a month Less than Once or twice At least 3-4 times Every day More than  once a week a week per week once a day | |
|  | |
| 1. **ever grind his/her teeth?** |  |
| Almost never Once a month Less than Once or twice At least 3-4 times Every day More than  once a week a week per week once a day | |
|  | |
| 1. **avoid (or try to avoid) certain objects or places?** |  |
| Almost never Once a month Less than Once or twice At least 3-4 times Every day More than  once a week a week per week once a day | |
|  | |
| 1. **ever take sharp intakes of breath or gasp?** |  |
| Almost never Once a month Less than Once or twice At least 3-4 times Every day More than  once a week a week per week once a day | |
|  | |
| 1. **vomit in response to specific situations (not illness)?** |  |
| Almost never Once a month Less than Once or twice At least 3-4 times Every day More than  once a week a week per week once a day | |
|  | |
| **Does he/she…** |  |
| 1. **ever look very worried or anxious?** |  |
| Almost never Once a month Less than Once or twice At least 3-4 times Every day More than  once a week a week per week once a day | |
|  | |
| 1. **ever look very sad or upset?** |  |
| Almost never Once a month Less than Once or twice At least 3-4 times Every day More than  once a week a week per week once a day | |
|  | |
| 1. **have an angry look on his/her face?** |  |
| Almost never Once a month Less than Once or twice At least 3-4 times Every day More than  once a week a week per week once a day | |
|  | |
| 1. **startle easily, or easily alarmed?** | |
| Almost never Once a month Less than Once or twice At least 3-4 times Every day More than  once a week a week per week once a day | |
|  | |
|  |  |
| **Over the past month, have you noticed…** |  |
| 1. **differences in the tone or pitch of his/her vocalisations that is not usual for him/her?** |  |
| Almost never Once a month Less than Once or twice At least 3-4 times Every day More than  once a week a week per week once a day | |
|  | |
| 1. **increased or different leg movements? (e.g. restlessness, tense, tremors, kicking, drawing legs up, jerking)?** |  |
| Almost never Once a month Less than Once or twice At least 3-4 times Every day More than  once a week a week per week once a day | |
|  | |
| 1. **that he/she needs to urinate or requests to use the toilet more than usual?** |  |
| Almost never Once a month Less than Once or twice At least 3-4 times Every day More than  once a week a week per week once a day | |
|  | |
| 1. **that he/she appears to visibly sweat more than usual?** |  |
| Almost never Once a month Less than Once or twice At least 3-4 times Every day More than  once a week a week per week once a day | |
|  | |
| 1. **that he/she shakes or trembles?** |  |
| Almost never Once a month Less than Once or twice At least 3-4 times Every day More than  once a week a week per week once a day | |
|  | |
|  |  |
| **Over the past month, have you noticed..** |  |
| 1. **his/her face look tense?** |  |
| Almost never Once a month Less than Once or twice At least 3-4 times Every day More than  once a week a week per week once a day | |
|  | |
| 1. **him/her frown more than usual?** |  |
| Almost never Once a month Less than Once or twice At least 3-4 times Every day More than  once a week a week per week once a day | |
|  | |
| **Over the past month, has he/she…** |  |
| 1. **been hitting, holding or touching a part of their body?** |  |
| Almost never Once a month Less than Once or twice At least 3-4 times Every day More than  once a week a week per week once a day | |
|  | |
|  |  |
| **Over the past month, has his/her…** |  |
| 1. **movements ever become jerky?** |  |
| Almost never Once a month Less than Once or twice At least 3-4 times Every day More than  once a week a week per week once a day | |
|  | |
| 1. **lips ever become tight, pout or quiver?** |  |
| Almost never Once a month Less than Once or twice At least 3-4 times Every day More than  once a week a week per week once a day | |
|  | |
|  |  |
| **Over the past month, has he/she…** |  |
| 1. **engaged in repetitive motor, hand or body movements? (e.g. rocking, hand flapping)** | **37b. Over the past one month, has this behaviour been…** |
| Almost never Once a month Less than Once or twice At least 3-4 times Every day More than  once a week a week per week once a day | Occurring a lot more than is typical of him/her |
|  | Occurring a bit more than is typical of him/her |
|  | Occurring at the same rate that is typical of him/her |
|  | Occurring a bit less than is typical of him/her |
|  | Occurring a lot less than is typical of him/her |
| **Does he/she…** |  |
| 1. **insist on having the same daily routine? (e.g. at home/school/work?)** | **38b. Over the past one month, has this behaviour been…** |
| Almost never Once a month Less than Once or twice At least 3-4 times Every day More than  once a week a week per week once a day | Occurring a lot more than is typical of him/her |
|  | Occurring a bit more than is typical of him/her |
|  | Occurring at the same rate that is typical of him/her |
|  | Occurring a bit less than is typical of him/her |
|  | Occurring a lot less than is typical of him/her |
|  |  |
| **Is he/she…** |  |
| 1. **showing checking behaviours? (e.g. doors and windows are closed, everything is just 'right', everything is straight)** | **39b. Over the past one month, has this behaviour been…** |
| Almost never Once a month Less than Once or twice At least 3-4 times Every day More than  once a week a week per week once a day | Occurring a lot more than is typical of him/her |
|  | Occurring a bit more than is typical of him/her |
|  | Occurring at the same rate that is typical of him/her |
|  | Occurring a bit less than is typical of him/her |
|  | Occurring a lot less than is typical of him/her |
| **Is he/she…** |  |
| 1. **repeating words, sounds or phrases over and over?** | **40b. Over the past one month, has this behaviour been…** |
| Almost never Once a month Less than Once or twice At least 3-4 times Every day More than  once a week a week per week once a day | Occurring a lot more than is typical of him/her |
|  | Occurring a bit more than is typical of him/her |
|  | Occurring at the same rate that is typical of him/her |
|  | Occurring a bit less than is typical of him/her |
|  | Occurring a lot less than is typical of him/her |
|  |  |
| **Does his/her…** |  |
| 1. **face appear pale? (e.g. sickly, grey or pasty)** |  |
| Almost never Once a month Less than Once or twice At least 3-4 times Every day More than  once a week a week per week once a day | |
|  | |
| 1. **face go red or look hot? (not in response to physical exercise or hot places/climates)** |  |
| Almost never Once a month Less than Once or twice At least 3-4 times Every day More than  once a week a week per week once a day | |
|  | |
| 1. **breathing ever become heavier or faster (not in response to physical exercise or hot places/climates)?** |  |
| Almost never Once a month Less than Once or twice At least 3-4 times Every day More than  once a week a week per week once a day | |
|  | |
| 1. **face ever scrunch up tightly (marked/obvious)?** |  |
| Almost never Once a month Less than Once or twice At least 3-4 times Every day More than  once a week a week per week once a day | |
|  | |
|  |  |
| **Has he/she…** |  |
| 1. **lost interest in activities that he/she used to enjoy?** |  |
| Almost never Less than a quarter Less than About half of More than half About three All of the  of the time half the time the time the time quarters of time  the time | |
|  | |
|  |  |
| **Is he/she…** |  |
| 1. **eating more than is typical for him/her?** |  |
| Almost never Less than a quarter Less than About half of More than half About three All of the  of the time half the time the time the time quarters of time  the time | |
|  | |
| 1. **eating less than is typical for him/her?** |  |
| Almost never Less than a quarter Less than About half of More than half About three All of the  of the time half the time the time the time quarters of time  the time | |
|  | |
| 1. **less able to concentrate on their day-to-day activities?** |  |
| Almost never Less than a quarter Less than About half of More than half About three All of the  of the time half the time the time the time quarters of time  the time | |
|  | |
|  |  |
| 1. **Has he/she lost weight?** |  |
| Yes No |  |
| 1. **Has he/she gained weight?** |  |
| Yes No |  |
|  |  |
| **Is he/she…** |  |
| 1. **fussy with food or have a restricted diet?** | **51b. Over the past one month, has this behaviour been…** |
| Almost never Less than a quarter Less than About half of More than half About three All of the  of the time half the time the time the time quarters of time  the time | Occurring a lot more than is typical of him/her |
|  | Occurring a bit more than is typical of him/her |
|  | Occurring at the same rate that is typical of him/her |
|  | Occurring a bit less than is typical of him/her |
|  | Occurring a lot less than is typical of him/her |
| **Does he/she…** |  |
| 1. **lack energy?** |  |
| Almost never Less than a quarter Less than About half of More than half About three All of the  of the time half the time the time the time quarters of time  the time | |
|  | |
| 1. **get tired for no apparent reason?** |  |
| Almost never Less than a quarter Less than About half of More than half About three All of the  of the time half the time the time the time quarters of time  the time | |
|  | |
| 1. **have a particular interest or a favourite object that has increased in intensity?** |  |
| Almost never Less than a quarter Less than About half of More than half About three All of the  of the time half the time the time the time quarters of time  the time | |
|  | |
| 1. **have a particular interest or a favourite object that has decreased in intensity?** |  |
| Almost never Less than a quarter Less than About half of More than half About three All of the  of the time half the time the time the time quarters of time  the time | |
|  | |
| 1. **seem unhappy or irritable?** |  |
| Almost never Less than a quarter Less than About half of More than half About three All of the  of the time half the time the time the time quarters of time  the time | |
|  | |
|  |  |
| **Over the past month, have you noticed…** |  |
| 1. **that he/she needs more help to look after themselves than they usually would? (e.g. they now need help washing where they used to do this alone)** |  |
| Almost never Less than a quarter Less than About half of More than half About three All of the  of the time half the time the time the time quarters of time  the time | |
|  | |
| 1. **any reduction in amount of his/her repetitive behaviour?** |  |
| Almost never Less than a quarter Less than About half of More than half About three All of the  of the time half the time the time the time quarters of time  the time | |
|  | |
|  |  |
| **Is he/she…** |  |
| 1. **having difficulty sleeping? (e.g. waking during the night, waking earlier in the morning)** | **59b.**  **Over the past one month, has this behaviour been…** |
| Almost never Less than a quarter Less than About half of More than half About three All of the  of the time half the time the time the time quarters of time  the time | Occurring a lot more than is typical of him/her |
|  | Occurring a bit more than is typical of him/her |
|  | Occurring at the same rate that is typical of him/her |
|  | Occurring a bit less than is typical of him/her |
|  | Occurring a lot less than is typical of him/her |
|  |  |
| **Is he/she…** |  |
| 1. **spending more time asleep than usual? (e.g. not waking in the morning, sleeping during the day)** | **60b.**  **Over the past one month, has this behaviour been…** |
| Almost never Less than a quarter Less than About half of More than half About three All of the  of the time half the time the time the time quarters of time  the time | Occurring a lot more than is typical of him/her |
|  | Occurring a bit more than is typical of him/her |
|  | Occurring at the same rate that is typical of him/her |
|  | Occurring a bit less than is typical of him/her |
|  | Occurring a lot less than is typical of him/her |
|  |  |
| **Is he/she…** |  |
| 1. **quiet and spending time alone?** | **61b.**  **Over the past one month, has this behaviour been…** |
| Almost never Less than a quarter Less than About half of More than half About three All of the  of the time half the time the time the time quarters of time  the time | Occurring a lot more than is typical of him/her |
|  | Occurring a bit more than is typical of him/her |
|  | Occurring at the same rate that is typical of him/her |
|  | Occurring a bit less than is typical of him/her |
|  | Occurring a lot less than is typical of him/her |
|  |  |
| **Do his/her…** |  |
| 1. **vocalisations sound slow or lack emotion?** | **62b.**  **Over the past one month, has this behaviour been…** |
| Almost never Less than a quarter Less than About half of More than half About three All of the  of the time half the time the time the time quarters of time  the time | Occurring a lot more than is typical of him/her |
|  | Occurring a bit more than is typical of him/her |
|  | Occurring at the same rate that is typical of him/her |
|  | Occurring a bit less than is typical of him/her |
|  | Occurring a lot less than is typical of him/her |
|  |  |
| **Does his/her…** |  |
| 1. **face appear expressionless or lacking emotion?** | **63b.**  **Over the past one month, has this behaviour been…** |
| Almost never Once a month Less than Once or twice At least 3-4 times Every day More than  once a week a week per week once a day | Occurring a lot more than is typical of him/her |
|  | Occurring a bit more than is typical of him/her |
|  | Occurring at the same rate that is typical of him/her |
|  | Occurring a bit less than is typical of him/her |
|  | Occurring a lot less than is typical of him/her |
|  |  |
| **How often are these statements true for your child/the person you support?** |  |
| 1. **Preparing him/her before things happen helps to reduce his/her distress** |  |
| Almost never Less than a quarter Less than About half of More than half About three All of the  of the time half the time the time the time quarters of time  the time | |
|  | |
| 1. **Removing the person I care for from a situation, or removing an item/object, generally calms them down** |  |
| Almost never Less than a quarter Less than About half of More than half About three All of the  of the time half the time the time the time quarters of time  the time | |
|  | |
| 1. **When the person I care for is distressed, I am able to calm or comfort him/her** |  |
| Almost never Less than a quarter Less than About half of More than half About three All of the  of the time half the time the time the time quarters of time  the time | |
|  | |
| 1. **When in certain preferred environments (e.g. home, their bedroom) the person I care for generally appears calm and relaxed** |  |
| Almost never Less than a quarter Less than About half of More than half About three All of the  of the time half the time the time the time quarters of time  the time | |
|  | |
| 1. **We are unable to do ‘typical’ day to day activities because of the emotional distress that would cause him/her (e.g. holidays, visiting friends, going for meals, general days out).** | |
| Almost never Less than a quarter Less than About half of More than half About three All of the  of the time half the time the time the time quarters of time  the time | |
|  | |
| 1. **We are unable to do activities we used to do with the person I care for because of the emotional distress he/she would experience.** | |
| Almost never Less than a quarter Less than About half of More than half About three All of the  of the time half the time the time the time quarters of time  the time | |
|  | |
| 1. **Have you noticed any other changes in behaviour or mood not covered in this questionnaire? If yes, please give details** |  |
|  |  |

*Items removed from the beta version of the ClASP-ID.*

| Item | Reason removed |
| --- | --- |
| 01. Has he/she been clinging to or seeking physical comfort from someone familiar? | Did not load to the final four factors in the initial EFA |
| 02.     Has he/she been asking repetitive questions? | Could not distinguish between those with and without a diagnosis of an anxiety disorder by t-test |
| 03.     Does he/she appear quiet and/or less active? | Could not distinguish between those with and without a diagnosis of an depression by t-test |
| 05. Does he/she throw himself/herself to the ground or thrash around? | Did not load to the final four factors in the initial EFA |
| 08. Does he/she ever become tense, stiff or rigid? | Dual loading above 0.4 |
| 09.     Does he/she ever appear more floppy than usual? | Could not distinguish between those in a high and low pain group by t-test |
| 15. Does he/she cry or well up as if he/she is about to cry? | Did not load to the final four factors in the initial EFA |
| 18.  Does he/she ever cover his/her eyes or ears? | Could not distinguish between those with and without a diagnosis of an anxiety disorder by t-test |
| 22. Does he/she vomit in response to specific situations (not illness)? | Did not load to the final four factors in the initial EFA |
| 24. Does he/she ever look very sad or upset? | Did not load to the final four factors in the initial EFA |
| 27. Over the past month, have you noticed differences in the tone or pitch of his/her vocalisations that is not usual for him/her? | Did not load to the final four factors in the initial EFA |
| 29. Over the past month, have you noticed that he/she needs to urinate or requests to use the toilet more than usual? | Did not load to the final four factors in the initial EFA |
| 30. Over the past month, have you noticed that he/she appears to visibly sweat more than usual? | Did not load to the final four factors in the initial EFA |
| 33. Over the past month, have you noticed him/her frown more than usual? | Did not load to the final four factors in the initial EFA |
| 37.  Over the past month, has he/she engaged in repetitive motor, hand or body movements? (e.g. rocking, hand flapping) | Did not load to the final four factors in the second EFA |
| 38. Does he/she insist on having the same daily routine? (e.g. at home/school/work?) | Cronbach’s alpha of the scale improved when removed |
| 39.  Is he/she showing checking behaviours? (e.g. doors and windows are closed, everything is just 'right', everything is straight) | Could not distinguish between those with and without a diagnosis of an anxiety disorder by t-test |
| 40.  Is he/she repeating words, sounds or phrases over and over? | Could not distinguish between those with and without a diagnosis of an anxiety disorder by t-test |
| 41.  Does his/her face appear pale? (e.g. sickly, grey or pasty) | Could not distinguish between those with and without a diagnosis of an anxiety disorder by t-test |
| 42. Does his/her face go red or look hot? (not in response to physical exercise or hot places/climates) | Did not load to the final four factors in the initial EFA |
| 43.  Does his/her breathing ever become heavier or faster (not in response to physical exercise or hot places/climates)? | Did not load to the final four factors in the second EFA |
| 44.  Does his/her face ever scrunch up tightly (marked/obvious)? | Poor test-retest reliability, of below 0.4 by spearman’s correlation |
| 46. Is he/she eating more than is typical for him/her? | Did not load to the final four factors in the initial EFA |
| 47. Is he/she eating less than is typical for him/her? | Did not load to the final four factors in the initial EFA |
| 48. Is he/she less able to concentrate on their day-to-day activities? | Did not load to the final four factors in the initial EFA |
| 49. Has he/she lost weight? | Did not load to the final four factors in the initial EFA |
| 50. Has he/she gained weight? | Did not load to the final four factors in the initial EFA |
| 51. Is he/she fussy with food or have a restricted diet? | Did not load to the final four factors in the initial EFA |
| 54. Does he/she have a particular interest or a favourite object that has increased in intensity? | Did not load to the final four factors in the initial EFA |
| 55. Does he/she have a particular interest or a favourite object that has decreased in intensity? | Did not load to the final four factors in the initial EFA |
| 56. Does he/she seem unhappy or irritable? | Did not load to the final four factors in the initial EFA |
| 57.  Over the past month, have you noticed that he/she needs more help to look after themselves than they usually would? (e.g. they now need help washing where they used to do this alone) | Could not distinguish between those with and without a diagnosis of an depression by t-test |
| 58. Over the past month, have you noticed any reduction in amount of his/her repetitive behaviour? | Did not load to the final four factors in the initial EFA |
| 59. Is he/she having difficulty sleeping? (e.g. waking during the night, waking earlier in the morning) | Did not load to the final four factors in the initial EFA |
| 62. Do his/her vocalisations sound slow or lack emotion? | Did not load to the final four factors in the initial EFA |
| 63. Does his/her face appear expressionless or lacking emotion? | Did not load to the final four factors in the initial EFA |
