# Supplementary material for: Development and psychometric properties of the Clinical Anxiety Scale for People with Intellectual Disabilities (ClASP-ID)
Source: J Neurodev Disord. 2024 Jul 27;16:43. doi: 10.1186/s11689-024-09554-9 (PMC11283710; doi:10.1186/s11689-024-09554-9)
Supplement: Supplementary file 4 — Additional file 4- Parent questionnaire feedback proforma. [file 11689_2024_9554_MOESM4_ESM.docx]

**Additional File 4- Parent Questionnaire Feedback Proforma**

**Questionnaire Feedback Document**

Listed below are the items we have created that will appear on our questionnaire. When completing the questionnaire, respondents will be asked to rate the items on a scale similar to this:

Almost never

Once or twice a week

At least 3-4 times per week

Every day

Less than once a week

More than once a day

Once a month

**The following instructions will be presented to participants:**

*This questionnaire is going to ask you about behaviours you may or may not have seen in the person you care for, over the last ONE MONTH. For each item, you will be asked to rate how frequently the behaviours have occurred over the last month. For some of the questions, we will also ask you to think about whether the behaviour is occurring more or less than is typical of the person you care for. Please try to answer every question. If you are unsure whether you have seen the behaviour, please select ‘almost never’ and move onto the next question.*

| **Is there anything about the instructions that is unclear? Do you have any feedback on the instructions?** |
| --- |

**The following items then appear on the questionnaire.**

**We are looking to know whether:**

- **Any of the items are confusing/unclear?**
- **There is a better way the question could be phrased?**

| **QUESTION** | **CONFUSING OR SUGGESTIONS FOR REPHRASING?** |
| --- | --- |
| 1. Has he/she been clinging to or seeking physical comfort from someone familiar? |  |
| 1. Has he/she been asking repetitive questions? |  |
| 1. Does he/she cry or have watery eyes? |  |
| 1. Does he/she ever freeze, stick to the spot or plant himself/herself to the ground? |  |
| 1. Does he/she appear quiet and/or less active? |  |
| 1. Has he/she seemed withdrawn or ‘vacant’? |  |
| 1. Does he/she throw himself/herself to the ground or thrash around? |  |
| 1. Does he/she ever make negative or frustrated vocalisations? (examples: whining, grumbling, growling, shouting, screaming) |  |
| 1. Does he/she appear on edge or on the lookout for danger? |  |
| 1. Do his/her movements ever become jerky? |  |
| 1. Does he/she ever become tense, stiff or rigid? |  |
| 1. Does he/she ever appear more floppy? |  |
| 1. Does he/she ever seem protective of a particular part of their body?   (e.g. holding it, guarding it, flinching) |  |
| 1. Have you noticed a change in their leg movements?   (e.g. restlessness, tense, tremors, kicking, drawing legs up, jerking) |  |
| 1. Has he/she been hitting, holding or touching a part of their body? |  |
| 1. Does he/she pace or jump around the room? |  |
| 1. Does he/she ever appear restless or agitated? |  |
| 1. Does he/she ever run away or hide from certain objects or situations? |  |
| 1. Does he/she ever cover him/herself with a blanket or try to place a barrier between him/herself and others? |  |
| 1. Is he/she engaging in repetitive motor, hand or body movements (e.g. rocking, hand flapping)? |  |
| 1. Does he/she insist on having the same daily routine (e.g. at home/school/work)? |  |
| 1. Is he/she showing checking behaviours (e.g. doors and windows are closed, everything is just ‘right’, everything is straight)? |  |
| 1. Is he/she repeating words, sounds or phrases over and over? |  |
| 1. Does he/she ever cover his/her eyes or ears? |  |
| 1. Does he/she ever grind his/her teeth? |  |
| 1. Does he/she avoid (or try to avoid) certain objects or places? |  |
| 1. Does he/she endure certain objects or situations despite showing signs of distress? |  |
| 1. Preparing him/her before things happen helps to reduce his/her distress? |  |
| 1. Removing the person I care for from a situation, or removing an item/object, generally calms them down. |  |
| 1. When the person I care for is distressed, I am able to calm or comfort him/her. |  |
| 1. When in certain *preferred* environments (e.g. home, their bedroom) the person I care for generally appears calm and relaxed. |  |
| 1. Does his/her face appear pale (sickly or pasty)? |  |
| 1. When distressed, does his/her face go red or look hot? |  |
| 1. Does he/she ever take sharp intakes of breath or gasp? |  |
| 1. Does his/her breathing ever become heavier or faster? |  |
| 1. When distressed, does he/she vomit? |  |
| 1. Have you noticed he/she has periods where his/her heart beats faster than usual? |  |
| 1. Have you noticed that he/she needs to urinate more than usual? |  |
| 1. Have you noticed that he/she appears to visibly sweat more than usual? |  |
| 1. Have you noticed that he/she shakes or trembles? |  |
| 1. Does he/she seem unhappy or irritable? |  |
| 1. Has he/she lost interest in activities that he/she used to enjoy? |  |
| 1. Is he/she eating more than is typical for him/her? |  |
| 1. Is he/she eating less than is typical for him/her? |  |
| 1. Has he/she lost weight? |  |
| 1. Has he/she gained weight? |  |
| 1. Is he/she fussy with food or have a restricted diet? |  |
| 1. Does he/she lack energy? |  |
| 1. Does he/she get tired for no apparent reason? |  |
| 1. Does he/she have a particular interest or a favourite object that has *increased* in intensity? |  |
| 1. Does he/she have a particular interest or a favourite object that has *decreased* in intensity? |  |
| 1. Is he/she able to concentrate on their day-to-day activities? |  |
| 1. Have you noticed that he/she needs *more* help to look after themselves than they usually would (e.g. they now need help washing where they used to do this alone)? |  |
| 1. Is he/she having difficulty sleeping (e.g. walking during the night, waking earlier in the morning)? |  |
| 1. Is he/she spending more time asleep than usual (e.g. not waking in the morning, sleeping during the day)? |  |
| 1. Is he/she quiet and spending time alone? |  |
| 1. Have you noticed any *reduction* in amount of his/her repetitive behaviour? |  |
| 1. Do his/her vocalisations sound slow or lack emotion? |  |
| 1. Have you noticed his/her face look tense? |  |
| 1. Does he/she ever look worried or anxious? |  |
| 1. Have you noticed he/she frown more than usual? |  |
| 1. Does he/she ever look sad or upset? |  |
| 1. Does his/her face ever scrunch up? |  |
| 1. Does he/she have an angry look on his/her face? |  |
| 1. Do his/her lips ever become tight, pout or quiver? |  |
| 1. Does his/her face appear expressionless or lacking emotion? |  |
| 1. Have you noticed any other changes in behaviour not covered in this questionnaire? |  |
| **Challenging Behaviour Supplement** | |
| 1. Does he/she hit, slap or kick other people? |  |
| 1. Does he/she scratch or bite other people? |  |
| 1. When distressed, does he/she lash out? |  |
| 1. Does he/she ever throw or bang objects? |  |
| 1. Does he/she damage items (not including accidents)? |  |
| 1. Does he/she display challenging behaviour whilst going to the toilet or just after going to the toilet (e.g. aggression or smearing)? |  |
| 1. Does he/she rub or pick at his/her skin, nails or wounds? |  |
| 1. Does he/she hit or slap his/her head or face? |  |
| 1. Does he/she bang his/her head off objects or the floor? |  |
| 1. Does he/she bite him/herself? |  |
| 1. Does he/she pull his/her own hair? |  |
| 1. Does he/she punch him/herself? |  |

**Thank you for taking the time to give feedback on our questionnaire items!**
