# Supplementary material for: Development and psychometric properties of the Clinical Anxiety Scale for People with Intellectual Disabilities (ClASP-ID)
Source: J Neurodev Disord. 2024 Jul 27;16:43. doi: 10.1186/s11689-024-09554-9 (PMC11283710; doi:10.1186/s11689-024-09554-9)
Supplement: Supplementary file 5 — Additional file 5- Demographic information for the inter-rater reliability and test-retest reliability samples. [file 11689_2024_9554_MOESM5_ESM.docx]

| *Demographic Information of the Inter-Rater Reliability Sample* | |
| --- | --- |
|  | N (%) |
| Person with ID Gender |  |
| Male | 12 (60.0) |
| Female | 8 (40.0) |
| Person with ID Age |  |
| Under 18 | 7 (35.0) |
| 18-24 | 5 (25.0) |
| 25-34 | 4 (20.0) |
| 35-44 | 4 (20.0) |
| 45-54 | 0 (0.0) |
| 55+ | 0 (0.0) |
| Intellectual Disability Diagnosis |  |
| Yes | 19 (95.0) |
| No | 1 (5.0) |
| Syndrome Diagnosis |  |
| Angelman Syndrome | 6 (30.0) |
| Cornelia de Lange Syndrome | 0 (0.0) |
| Fragile X Syndrome | 3 (15.0) |
| Prader-Willi Syndrome | 4 (20.0) |
| Down Syndrome | 0 (0.0) |
| Cri du Chat Syndrome | 1 (5.0) |
| Potocki-Lupski Syndrome | 1 (5.0) |
| Tuberous Sclerosis | 1 (5.0) |
| 9q34 Deletion | 0 (0.0) |
| Phelan McDermid Syndrome | 1 (5.0) |
| Pitt Hopkins Syndrome | 0 (0.0) |
| Other | 1 (5.0) |
| Psychiatric and/or Neurodevelopmental Diagnosis |  |
| Autism | 5 (25.0) |
| Anxiety | 2 (10.0) |
| ADHD | 3 (15.0) |
| Obsessive-Compulsive Disorders | 0 (0.0) |
| Depression | 2 (10.0) |
| Verbal Ability^a^ |  |
| Non-verbal | 6 (30.0) |
| Odd words only | 8 (40.0) |
| Fully verbal | 6 (30.0) |
| Can talk but doesn’t | 0 (0.0) |
| Self-Help^a^ |  |
| Not able | 9 (45.0) |
| Partly able | 3 (15.0) |
| Able | 8 (40.0) |
| Mobility^a^ |  |
| Non-ambulant | 2 (10.0) |
| Partly mobile | 5 (25.0) |
| Fully mobile | 13 (65.0) |
| a Information obtained using the Wessex Questionnaire (Kushlick, Blunden & Cox, 1979) | |

**Additional File 5- Demographic Information for Inter-Rater and Test-Retest Reliability Samples**

| *Demographic Information of the Test-Retest Reliability Sample* | |
| --- | --- |
|  | N (%) |
| Person with ID Gender |  |
| Male | 47 (60.3) |
| Female | 31 (39.7) |
| Person with ID Age |  |
| Under 18 | 27 (34.6) |
| 18-24 | 19 (24.4) |
| 25-34 | 13 (16.7) |
| 35-44 | 18 (23.1) |
| 45-54 | 0 (0.0) |
| 55+ | 1 (1.3) |
| Intellectual Disability Diagnosis |  |
| Yes | 74 (94.9) |
| No | 4 (5.1) |
| Syndrome Diagnosis |  |
| Angelman Syndrome | 15 (19.2) |
| Cornelia de Lange Syndrome | 12 (15.4) |
| Fragile X Syndrome | 6 (7.7) |
| Prader-Willi Syndrome | 6 (7.7) |
| Down Syndrome | 3 (3.8) |
| Cri du Chat Syndrome | 3 (3.8) |
| Potocki-Lupski Syndrome | 4 (5.1) |
| Tuberous Sclerosis | 2 (2.6) |
| 9q34 Deletion | 1 (1.3) |
| Phelan McDermid Syndrome | 1 (1.3) |
| Pitt Hopkins Syndrome | 1 (1.3) |
| Other | 5 (6.4) |
| Psychiatric and/or Neurodevelopmental Diagnosis |  |
| Autism | 27 (34.6) |
| Anxiety | 12 (15.4) |
| ADHD | 7 (9.0) |
| Obsessive-Compulsive Disorders | 3 (3.8) |
| Depression | 2 (2.6) |
| Verbal Ability^a^ |  |
| Non-verbal | 23 (29.5) |
| Odd words only | 28 (35.9) |
| Fully verbal | 26 (33.3) |
| Can talk but doesn’t | 1 (1.3) |
| Self-Help^a^ |  |
| Not able | 28 (35.9) |
| Partly able | 30 (38.5) |
| Able | 20 (25.6) |
| Mobility^a^ |  |
| Non-ambulant | 12 (15.4) |
| Partly mobile | 20 (25.6) |
| Fully mobile | 46 (59.0) |
| a Information obtained using the Wessex Questionnaire (Kushlick, Blunden & Cox, 1979) | |
