# Supplementary material for: Development and psychometric properties of the Clinical Anxiety Scale for People with Intellectual Disabilities (ClASP-ID)
Source: J Neurodev Disord. 2024 Jul 27;16:43. doi: 10.1186/s11689-024-09554-9 (PMC11283710; doi:10.1186/s11689-024-09554-9)
Supplement: Supplementary file 6 — Additional file 6- Further psychometric properties of the ClASP-ID, including internal consistency and test-retest reliability of specific sample subgroups. [file 11689_2024_9554_MOESM6_ESM.docx]

**Additional File 6- Further Psychometric Properties of the ClASP-ID**

*Internal consistency of the ClASP-ID by different subgroups in the sample.*

|  | *n* | Anxiety | Pain | Low Mood | Consolability |
| --- | --- | --- | --- | --- | --- |
| Total | 311 | .921 | .812 | .809 | .630 |
| Children | 135 | .907 | 837 | .816 | .613 |
| Adults | 175 | .923 | .769 | .802 | .638 |
| Non-syndromic autism | 98 | .880 | .835 | .828 | .657 |
| All autism | 156 | .905 | .827 | .803 | .621 |
| Minimally verbal^a^ | 211 | .907 | .780 | .764 | . 629 |
| Non-verbal^a^ | 81 | .913 | .829 | .819 | .576 |
| Minimally verbal and not able^a^ | 105 | .914 | .794 | .798 | .631 |
| Non- or partially ambulant^a^ | 123 | .919 | .827 | .846 | .639 |
| a Information obtained using the Wessex Questionnaire (Kushlick, Blunden & Cox, 1979) | | | | | |

*Test-retest reliability of the ClASP-ID by different subgroups in the sample.*

|  | *n* | Anxiety | Pain | Low Mood | Consolability |
| --- | --- | --- | --- | --- | --- |
| Total | 78 | .881 | .797 | .785 | .670 |
| Children | 27 | .871 | .627 | .780 | .778 |
| Adults | 51 | .882 | .869 | .791 | .612 |
| All autism | 27 | .826 | .808 | .814 | .729 |
| Minimally verbal^a^ | 26 | .911 | .828 | .867 | .636 |

a Information obtained using the Wessex Questionnaire (Kushlick, Blunden & Cox, 1979)
